# Effects of GC Bias in Next-Generation-Sequencing Data on *De Novo* Genome Assembly

**DOI:** 10.1371/journal.pone.0062856

**Published:** 2013-04-29

**Authors:** Yen-Chun Chen, Tsunglin Liu, Chun-Hui Yu, Tzen-Yuh Chiang, Chi-Chuan Hwang

**Affiliations:** 1 Department of Engineering Science, National Cheng Kung University, Tainan, Taiwan; 2 Institute of Bioinformatics and Biosignal Transduction, National Cheng Kung University, Tainan, Taiwan; 3 Department of Life Sciences, National Cheng Kung University, Tainan, Taiwan; 4 Supercomputing Research Center, National Cheng Kung University, Tainan, Taiwan; University of Georgia, United States of America

## Abstract

Next-generation-sequencing (NGS) has revolutionized the field of genome assembly because of its much higher data throughput and much lower cost compared with traditional Sanger sequencing. However, NGS poses new computational challenges to *de novo* genome assembly. Among the challenges, GC bias in NGS data is known to aggravate genome assembly. However, it is not clear to what extent GC bias affects genome assembly in general. In this work, we conduct a systematic analysis on the effects of GC bias on genome assembly. Our analyses reveal that GC bias only lowers assembly completeness when the degree of GC bias is above a threshold. At a strong GC bias, the assembly fragmentation due to GC bias can be explained by the low coverage of reads in the GC-poor or GC-rich regions of a genome. This effect is observed for all the assemblers under study. Increasing the total amount of NGS data thus rescues the assembly fragmentation because of GC bias. However, the amount of data needed for a full rescue depends on the distribution of GC contents. Both low and high coverage depths due to GC bias lower the accuracy of assembly. These pieces of information provide guidance toward a better *de novo* genome assembly in the presence of GC bias.

## Introduction

Genome sequencing and assembly are essential for understanding the secrets behind genomes. Next-generation-sequencing (NGS) has revolutionized the field of genomics [1], [2] since its recent appearance [3] because of its much higher data throughput, thus much lower cost, and the much faster speed compared with traditional Sanger sequencing [4]. As a result, the number of genome projects increases at an un-precedent pace [5], and is expected to keep increasing because the current number of complete genomes is only a tiny fraction of the number of species on earth.

Despite the great advantages of NGS, genome assembly using NGS data is challenging for several reasons [6], [7], among those sequencing bias in NGS data has been a known issue [8]. On Illumina system [9], a major NGS platform, it has been reported that extreme base compositions, i.e., GC-poor or GC-rich sequences, lead to an uneven coverage or even no coverage of reads across the genome [9], [10], [11], [12], [13]. For example, Illumina sequencing of a *Plasmodium falciparum* genome, which is extremely GC-poor with a mean GC content less than 25%, was found to favor the more GC-balanced regions, leading to few or no reads from the many GC-poor regions [13]. As read coverage is a crucial piece of information exploited by many assemblers, uneven coverage or no coverage surely hinders genome assembly. Most current assemblers, except Velvet-SC [14], assume a uniform coverage of reads across genomes during assembly. Low coverage regions may be considered as the results of sequencing errors and neglected. On the other hand, high coverage regions may be treated as repetitive elements, leading to assembly fragmentations. Using the extremely GC biased Illumina data of *P. falciparum* mentioned above, an assembly was even not possible [13].

Uneven coverage of reads resulted from GC bias can be introduced at several processes of Illumina sequencing, e.g., PCR amplification of library, cluster amplification, and the sequencing step [15]. Different sequencing kits and protocols also contribute to the variation in the nature and degree of GC bias. Among these factors, library amplification by PCR plays a major role in generating GC bias [15]. New experimental designs, e.g., amplification-free Illumina sequencing [13] and optimized PCR protocols [15], [16], have been proposed to reduce GC bias. In these studies, the depletions of reads in GC-poor and GC-rich regions were greatly compensated. However, it is not clear how the new experimental designs apply in general. The amplification-free approach requires a greater amount of starting DNA materials, thus less applicable when the amount of samples is limited [16]. As bias is known to vary between laboratories and from run to run, the optimized PCR protocols may not be successfully carried out. All these factors result in GC bias at various degrees in real Illumina data.

Although GC bias is known to aggravate genome assembly, it is not clear to what extent an assembly is made worse at varying degrees of GC bias. The performance of assemblers on NGS data has been explored in a few studies [17], [18]. However, none of these works discusses the effects of GC bias on genome assembly. Lin et al. study the performance of genome assembly using Illumina data simulated from few genomes of low or high mean GC content [19]. However, they do not introduce GC bias during simulation. Facing the various degrees of GC bias in real NGS data, two pieces of information are crucial for assembly: the degree of GC bias in real NGS data and the extent that an assembly is affected by that degree of GC bias. To obtain such information, we conducted a systematic analysis on the effects of GC bias on genome assembly. The obtained quantitative knowledge provides guidance to the experimental design toward a better genome assembly.

In this study, we estimated the degree of GC bias in fourteen real Illumina data sets from six genomes. The results revealed a wide range of degrees of GC bias among the data sets. We then simulated Illumina paired-end (PE) reads at various degrees of GC bias and evaluated the resulting genome assemblies. Illumina PE reads were simulated from three bacterial and two plant genomes, which data were then assembled by eight popular assemblers. With these results, we revealed many relationships between the degree of GC bias and the completeness and accuracy of assembly.

## Materials and Methods

### Genomic Sequences and NGS Data

To assess GC bias in real NGS data, we downloaded from NCBI Sequence Read Archive database [20] fourteen Illumina libraries of six bacterial genomes (Table 1), as well as their genomic sequences from NCBI Genome database. For studying the impacts of GC bias on genome assembly, we simulated PE libraries (see below) from the genomic sequences of three bacterial and two plant genomes (Table 2), which were also downloaded from NCBI.

**Table 1:** Genomes and Illumina libraries for studying GC bias in real NGS data.

Species	Genome(accession ID)	NGS data accession ID(read length, insert length,and window size for GCcontent calculation)	MeanGC content	GC Std.	GC bias (slope)	polymerase and PCR cycles
*Pseudomonas fluorescens Pf0-1*	NC_007492.2	DRR001171 (91, 397 and 397 bp)	60.5%	4.5%	−1.96	Phusion DNA with 18 cycles†
*Shewanella amazonensis SB2B*	NC_008700.1	SRR090701 (76, N.A. and 200 bp)	53.6%	5.3%	3.41	Phusion DNA with 10 or 12 cycles*
*Escherichia coli K-12 MG1655*	NC_000913.2	SRR001666 (36, 339 and 339 bp)	50.8%	5.8%	−0.07	Phusion DNA with 10 or 12 cycles*
*Escherichia coli K-12 MG1655*	NC_000913.2	SRR350605 (76, 224 and 224 bp)	50.8%	6.3%	−1.9	Phusion DNA with 10 or 12 cycles*
*Escherichia coli K-12 MG1655*	NC_000913.2	SRR398955 (76, 229 and 229 bp)	50.8%	6.3%	−1.55	Phusion DNA with 10 or 12 cycles*
*Escherichia coli K-12 MG1655*	NC_000913.2	SRR402738 (76, 225 and 225 bp)	50.8%	6.3%	−2.6	Phusion DNA with 10 or 12 cycles*
*Staphylococcus aureus USA300*	NC_010079.1	SRR022866 (76, 171 and 171 bp)	32.8%	5.3%	−5.3	Pfu Ultra II Fusion HS DNA with totally 26 cycles**
*Staphylococcus aureus USA300*	NC_010079.1	SRR022867 (36, 162 and 162 bp)	32.8%	5.3%	−4.49	Pfu Ultra II Fusion HS DNA with totally 26 cycles**
*Staphylococcus aureus USA300*	NC_010079.1	SRR022868 (101, 171 and 171 bp)	32.8%	5.3%	−5.05	Pfu Ultra II Fusion HS DNA with totally 26 cycles**
*Staphylococcus aureus MRSA252*	NC_002952.2	SRR342227 (101, 207 and 207 bp)	32.8%	5.0%	4.13	N.A.
*Mycobacterium tuberculosis* *H37Rv*	NC_000962.2	SRR099031 (76, 174 and 174 bp)	65.6%	5.0%	−1.1	N.A.
*Mycobacterium tuberculosis* *H37Rv*	NC_000962.2	SRR017680 (51, N.A. and 200 bp)	65.6%	4.8%	−5.24	N.A.
*Mycobacterium tuberculosis* *H37Rv*	NC_000962.2	SRR023440 (76, 193 and 193 bp)	65.6%	4.9%	−8.86	N.A.
*Mycobacterium tuberculosis* *H37Rv*	NC_000962.2	SRR023441 (76, 195 and 195 bp)	65.6%	4.9%	−8.96	N.A.

Except the two libraries, SRR090701 and SRR017680, all libraries are PE reads.

† The information is from Illumina Multiplexing Sample Preparation Guide as mentioned by Shintani et al. [42].

* The information is from Illumina Paired-End Sequencing Sample Preparation Guide as described in the NCBI SRA database.

** The information is from Fisher et al. [43].

**Table 2:** Genomic sequences for simulating PE libraries with GC bias.

Species	NCBI accessions	Size (Mb)	Mean GC (%)	GC Std.* (%)
*Staphylococcus aureus*	NC_010079.1	2.87	32.8	5.2
*Escherichia coli*	NC_000913.2	4.64	50.8	6.5
*Mycobacterium tuberculosis*	NC_000962.2	4.41	65.6	5
*Oryza sativa* Chr. 5	NC_008398.2	30.04	44	12.6
*Arabidopsis thaliana* Chr.1	NC_003070.9	30.43	35.9	7.6

* The standard deviation of GC contents is calculated using a 180-bp window.

### Quantification of GC Bias

To explore GC bias in real NGS data, we aligned Illumina PE reads to the reference genome by Novoalign [21], which was selected because of its good performance in general [22]. We demanded Novoalign to report all alignments of a read if it has multiple hits to the reference. Novoalign calculated fragment length and reported the mean value, which was later used for quantifying GC bias. We obtained the coverage depths across the genome by parsing the alignment results.

**Figure 1:**
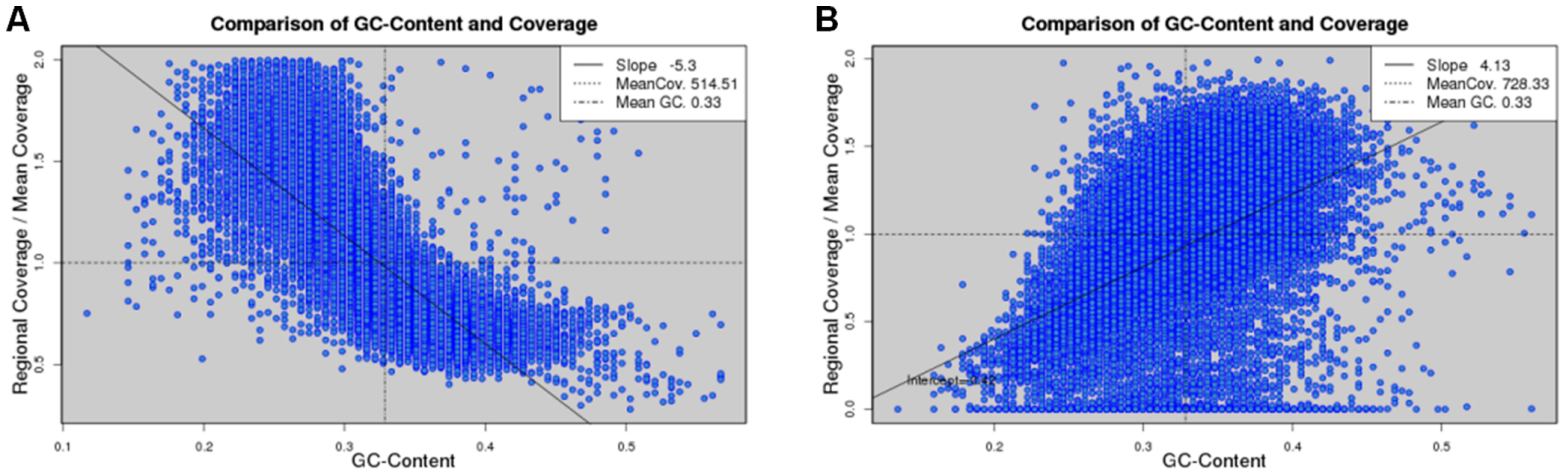
Scatter plots of GC content and read coverage of real Illumina data. The data sets are from *S. aureus* USA300 (A) and *S. aureus* MRSA252 (B) genomes. Read coverage is normalized to the mean value, which is represented by a horizontal dashed line. A vertical dashed line denotes the mean GC content. The data points are fitted by a straight line and the slope is defined as the degree of GC bias. The two cases represent a negative and positive GC bias, respectively.

To study the relationship between GC bias and read coverage, we scanned a genome with a sliding window of size equal to the mean fragment length and the step size was set as half the window size. In each window, we calculated the GC content, i.e., the percentage of G and C bases in the window, as well as the average read coverage. This resulted in many data points of GC contents and read coverage (see Figure 1 for an example). Read coverage was normalized to the mean value so that the results would not scale with the amount of data. We eliminated the data points whose coverage was more than twice the mean coverage because they likely represented repeats. We fit the remaining data points by a straight line and defined the slope as the degree of GC bias in the real data.

### Simulation of Illumina PE Library

Sequencing data is considered as GC biased if more (or less) reads tend to come from a region with a higher GC content. To simulate PE reads with a GC bias, we first defined the probability of generating a DNA fragment of certain GC content from a genome. Formula one describes that the probability, P, of generating a DNA fragment, F, is proportional to the GC content of the DNA fragment, GC(F). Specifically, it calculates the difference between GC(F) and the mean GC content, GC_m_, according to which it sets the deviation of the generation probability from the mean value. The slope, s, is defined as the degree of GC bias in this work. The constant C is a normalization factor. This probability is used to generate PE reads with a linear GC bias in a random manner.
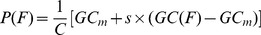
(1)


In addition to GC bias, our simulation took into account position-dependent error profiles and the distribution of insert lengths as in the real NGS data. From the quality scores of real Illumina reads, we calculated the mean error rate at each base position of reads, which was then used to introduce errors in the simulated reads. Insert lengths were simulated to follow a normal distribution with a mean value of 180 bp and a standard deviation of 10 bp. Under these criteria, we simulated PE reads of length 100 bp to a 50X coverage or more.

Our simulation ran in the following steps: (i) collecting all possible DNA fragments of size 180 bp from the genome, i.e., a DNA fragment starting at each base position is generated, (ii) calculating the GC content for each DNA fragment and the mean value of all DNA fragments, (iii) randomly selecting a starting position and generating a DNA fragment length following a normal distribution, (iv) deciding to keep the DNA fragment or not according to the probability calculated from its GC content using formula one, (v) repeating (iii) and (iv) until the amount of reads reached a desired coverage e.g., 50X or 100X, (vii) extracting read pairs, each of length 100 bp, from the two ends of the DNA fragments, (vi) distributing errors into both reads according to the error profiles in a random fashion.

Since ALLPATHS-LG requires mate-pair reads for assembly, we further simulated a MP library without a GC bias at one fold coverage with a mean insert length 3.5 Kb and a standard deviation 500 bp for assembly by ALLPATHS-LG.

### Finding Repeats in Genome

We defined repeats as the duplicated sequences in a genome which length is larger than the mean insert length. We used PALS, a local alignment tool adopted by PILER [23], to detect repeats in a genome. By default, PALS reports an alignment when the identity is above 94%.

### Genome Assembly

In this study, we applied seven NGS assemblers: ALLPATHS-LG [24], [25], ABySS [26], Edena [27], SOAPdenovo [28], SSAKE [29], Velvet [30], [31] and Velvet-SC [14]. Velvet-SC is an extension of Velvet, designed to treat the sequencing data from a single cell, which is highly non-uniform. SOAPdenovo comes with a package called GapCloser, which further increases the completeness of assembly. However, the performance of GapCloser is not clear in general. In this study, we used SOAPdenovo without and with GapCloser (denoted by SOAP+GC), and said that we used eight assemblers.

Because assembly can be greatly affected by parameters, we optimized assembly via scanning possible values for crucial parameters. Four assemblers, ABySS, SOAPdenovo, Velvet, and Velvet-SC take the de-Bruijn graph approach, which sets a crucial parameter, k-mer, during assembly. For the four assemblers, we scanned through possible k-mer values for the largest N50 length of contigs. Edena and SSAKE allow users to set the minimum overlap between reads during assembly. Again, we tried various minimum overlaps to obtain the optimized contig N50 length. For other assemblers, we used the default parameters. Note that we eliminated the contigs shorter than 100 bp from all the assemblies for a fair comparison.

### Mis-assembly

We applied GAGE [32] to detect errors in the assembled contigs. GAGE aligns contigs to the reference genome using NUCMER [33], which outputs several types of inconsistencies that are likely mis-assemblies. These include regions of INDELs, collapse of tandem repeats, unaligned reference sequences, relocation, inversion, translocations, and SNPs. We parsed these pieces of information from the intermediate outputs of GAGE, “out.rdiff”, “out.1delta”, “out.mdelta” and “out.snps”. The acquired data were then plotted using R package.

## Results

### GC Bias in Real NGS Data

We set out to explore the extent of GC bias in fourteen real Illumina data sets from six bacterial genomes (Table 1). GC bias describes the relationship between GC content and read coverage across a genome. That is, a genomic region of a higher GC content tends to have more (or less) Illumina reads covering that region. We aligned Illumina PE reads to the reference genome (Methods) and obtained the read coverage, i.e., number of reads covering each base position, across the genome. From the alignments of PE reads, the fragment lengths of the PEs were calculated. The GC content in a genomic region was defined as the percentage of G and C bases in that region. We scanned a genome with a sliding window of size equal to the mean fragment length and the step size was set as half the window size. In each window, we calculated the GC content and mean coverage of reads. These data resulted in a scatter plot of GC content and read coverage (see Figure 1 for an example).

Figure 1A and 1B show the relationship between GC content and read coverage in the real Illumina data of two bacterial genomes, *S. aureus* USA300 (data set SRR022866) and *S. aureus* MRSA252 (SRR342227), respectively. We observed from the figures clear non-random relationships between GC content and read coverage in the real Illumina data from the two genomes. That is, the read coverage in a region of a higher GC content tended to be lower and higher in the cases of *S. aureus* USA300 and *S. aureus* MRSA252, respectively, which illustrated the presence of GC bias in both cases. To quantify GC bias, we fit the data points of GC content and read coverage with a straight-line, and defined the degree of GC bias as the slope of the fitted line (Methods). Note that the slope should not scale with the amount of data because the read coverage had been normalized to the mean coverage. In this definition, we found a negative and positive GC bias in the real data from the genomes of *S. aureus* USA300 and *S. aureus* MRSA252, respectively. The GC contents in the two bacterial genomes are similar (Table 1) since they are two strains of the same species. The distinct signs of GC bias in the two cases indicate that GC bias is not solely determined by genomic content.

**Figure 2:**
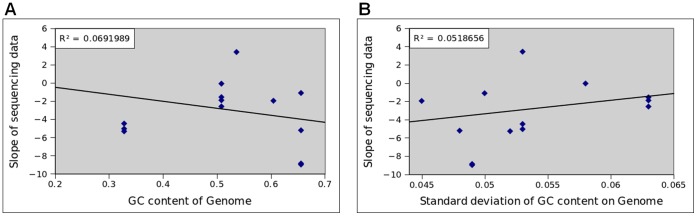
Correlation between the degree of GC bias and two statistics of GC contents. No correlation can be observed between the degree of GC bias (y-axis) and either the mean GC content (A) or the standard deviation of GC contents (B).

We repeated the above analysis and calculated the degrees of GC bias in twelve other sets of Illumina data. From Table 1 and Figure S1, we found that the degree of GC bias varied in those data, ranging from −8.9 (*M. tuberculosis*, SRR023441) to +4.1 (*S. aureus* MRSA252, SRR342227). In these data sets, the degree of GC bias did not correlate neither with the mean GC content nor with the standard deviation of GC content of the genome (Figure 2). These again indicate that genomic content itself does not determine GC bias.

### Simulation of Illumina PE Data with a GC Bias

**Figure 3:**
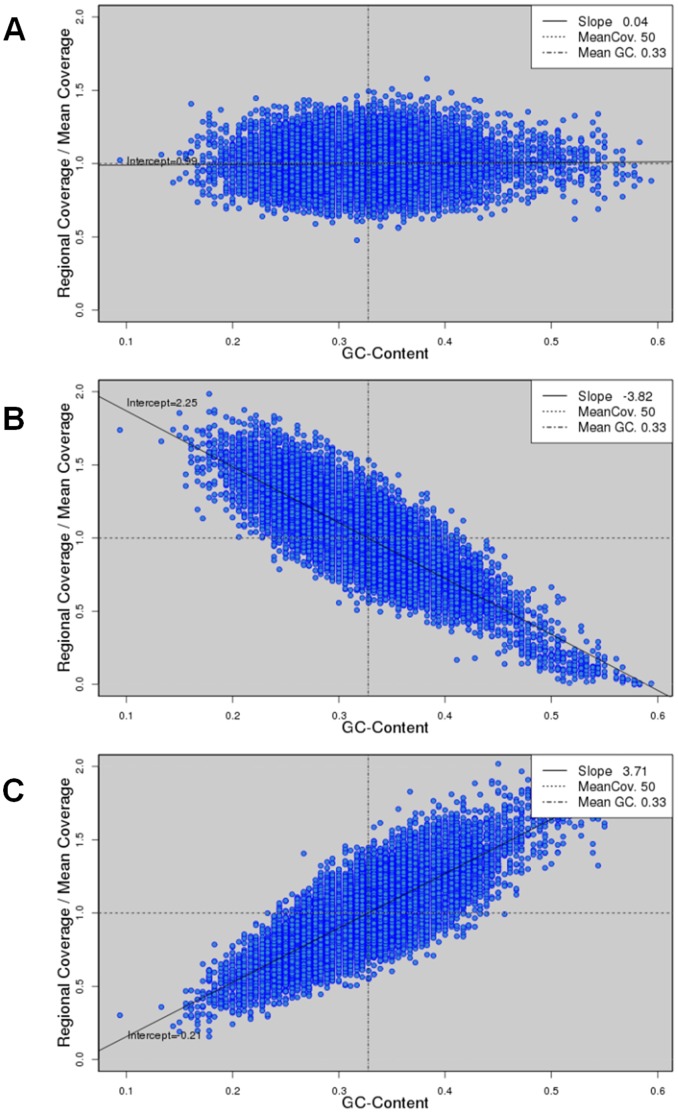
Scatter plots of GC content and read coverage of the simulated data. From the *S. aureus* USA300 genome, we simulated reads of 50X coverage at three degrees of GC bias: negative slope −3.83 (A), slope zero (B), and positive slope 3.72 (C).

To explore the effects of GC bias on *de novo* genome assembly, we resorted to simulated data. We obtained the complete genomes of three bacterial species (Table 2), and simulated PE reads with a linear GC bias at various degrees (Methods). The mean GC contents of the three bacteria, *S. aureus*, *E. coli*, and *M. tuberculosis*, are relatively low (32.8%), medium (50.8%), and high (62.6%), respectively (Table 2). From each genome, we simulated PE data at nine different degrees of GC bias (slope ranging from −3.8 to 3.8) covering the extent of GC bias in most of the real NGS data. The simulated reads were of length 100 bp, and constituted 50X coverage of the corresponding genomes. Figure 3 and Figure S2 shows the scatter plots of GC content versus read coverage in those simulated data sets. To explore the effects of GC bias on the assembly of more complex species, we included two plants, *A. thaliana* and *O. sativa* (Table 2). Chromosome one and five of the *A. thaliana* and *O. sativa* genomes, which GC contents are relatively low (35.9%) and slightly low (44%), were taken for simulation, respectively. For each chromosome, we simulated PE reads of length 100 bp to 100X coverage (Figure S3).

### Effects of GC Bias on the Completeness of Bacterial Genome Assemblies

For each bacterial genome, we assembled the simulated PE reads at nine different degrees of GC bias using eight assemblers (Methods). These assemblers cover three categories of assembly strategies: de-Bruijn graph, Overlap-Layout-Consensus, and greedy extension approach. ABySS, ALLPATHS-LG, SOAPdenovo, SOAP+GC, Velvet, and Velvet-SC take the de-Bruijn graph approach for assembly. SOAP+GC applies the GapCloser package after SOAPdenovo assembly. Velvet-SC is a version of Velvet proposed to assemble data with non-uniform coverage across the genome. Edena and SSAKE apply the Overlap-Layout-Consensus and greedy-extension approach for assembly, respectively. To consider the accuracy of assembly, we corrected errors in the assembled contigs using GAGE. Briefly, GAGE aligns contigs to the reference genome and detects assembly errors, e.g., SNPs, INDELs, translocations, … etc. It breaks contigs at every mis-join and every INDEL longer than 5 bp, and also outputs the statistics of the corrected contigs. The most common measure of assembly completeness is N50 length, defined so that 50% of the bases are in the contigs of length at least this value. We used the N50 length of corrected contigs to evaluate the completeness of assembly.

**Figure 4:**
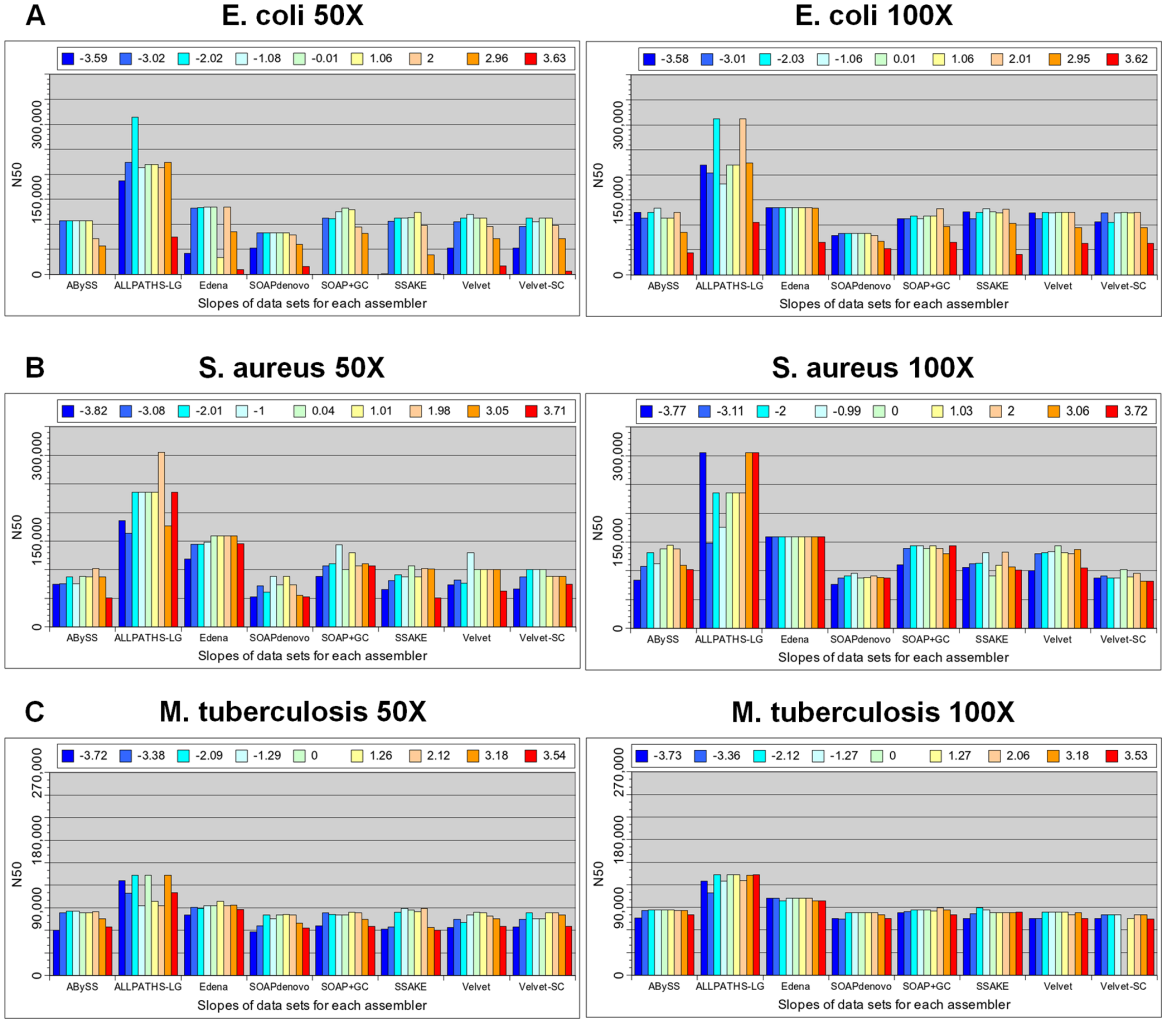
Completeness of assemblies of three bacterial genomes by eight assemblers, each treating nine data sets. The nine data sets at various degrees of GC bias (shown in different colors) are simulated from the genomes of three bacteria: *E. coli* (A), *S. aureus* (B), and *M. tuberculosis* (C). Assembly completeness is measured by the N50 length of the contigs after error corrections. The left and right columns show the results of assembly using simulated data of a 50X and 100X coverage, respectively. Note that the Velvet-SC assembly of the genome *M. tuberculosis* failed without a clear reason. At a 50X coverage, strong GC bias leads to more fragmented assembly in all cases. Such performance drops can be rescued via increasing the amount of data to a 100X coverage.

The left column of Figure 4A shows the corrected N50 lengths of the *E. coli* assemblies by the eight assemblers, each treating nine simulated data sets at various degrees of GC bias. For all assemblers, we found that weak GC bias, slope between −2 and 2, did not alter much the completeness of assembly in general. Among the nine assemblies by every assembler, the best corrected N50 length occurred in one of the data sets with a zero or weak GC bias. As GC bias got stronger, the corrected N50 length started to decrease in all cases, i.e., no assembler could maintain its completeness of assembly at a strong GC bias. At a strong positive GC bias, slope 3.7, the corrected N50 length dropped from the best value by about an order of magnitude or more for all assemblers except ALLPATHS-LG, where the corrected N50 length only dropped to about one-third of the best value.

We repeated the above analyses for the two other bacterial genomes, *S. aureus* and *M. tuberculosis*. For both genomes, we observed a similar trend that for all assemblers, a strong GC bias resulted in a more fragmented assembly compared with the results at no or weak GC bias (left column of Figure 4B and 4C). However, at a strong GC bias, the assembly fragmentations of the two genomes were not as serious as those of the *E. coli* genome for all assemblers. This indicates that the effects of GC bias on assembly completeness are species or genome dependent.

Note that for all simulated data of the three bacterial species, ALLPATHS-LG performed better in general. This was the result of the additional MP library for ALLPATHS-LG assembly. We reduced the amount of MP data from 1X to 20 read pairs and repeated the ALLPATHS-LG assemblies. With only 20 pairs of reads, the performances of ALLPATHS-LG were comparable to other assemblers (Figure S4). We emphasized that we did not intend to compare the performance of various assemblers in this work. Instead, we focused on comparing the assemblies by the same assembler at different degrees of GC bias.

### Effects of GC Bias versus the Amount of Data

It is known that assembly completeness can be affected significantly by several factors, e.g., data amount and assembly parameters. During assembly, we had explored the effects of key parameters, i.e., kmer value and overlap length (Methods). We scanned a range of parameter values and picked the assembly with the largest N50 length for comparisons. Here, we explored whether the amount of data altered the effects of GC bias on the completeness of assembly. For each of the three bacterial genomes, we simulated PE reads to 100X coverage, again at nine degrees of GC bias, for assembly. The results were then compared with those at a 50X coverage.

For *S. aureus* and *M. tuberculosis*, the effects of GC bias on the completeness of assembly disappeared in all cases except the slightly more fragmentations of the ABySS and Velvet assemblies of the *S. aureus* genome at a strong GC bias (right column of Figure 4B and 4C). This demonstrates that the effects of GC bias on assembly completeness can be rescued via supplying more data. Note that for every assembler, the best corrected N50 length among the nine data sets was not altered much when the amount of data was increased from 50X to 100X. This indicates that 50X coverage of reads with no or weak GC bias is already “enough” for achieving an optimal assembly. Thus, the extra amounts of data only serve to rescue the assembly fragmentations due to a strong GC bias.

For *E. coli*, the extra amount of data also rescued the assembly fragmentation because of GC bias in all cases, but did not achieve a full recovery (right column of Figure 4A). At 100X read coverage and a strong positive GC bias (slope 3.6), the corrected N50 lengths by all assemblers were 30.8% (by SSAKE)∼63.4% (by SOAPdenovo) of the best values at a zero or weak GC bias. The partial rescues motivated us to explore whether assembly fragmentation because of GC bias can be fully rescued if the amount of data keeps increasing. To answer this question, we further simulated from the *E. coli* genome PE reads with no GC bias and a strong positive GC bias, each of 250X, 500X, 1000X, and 2000X coverage, for assembly by all eight assemblers.

**Figure 5:**
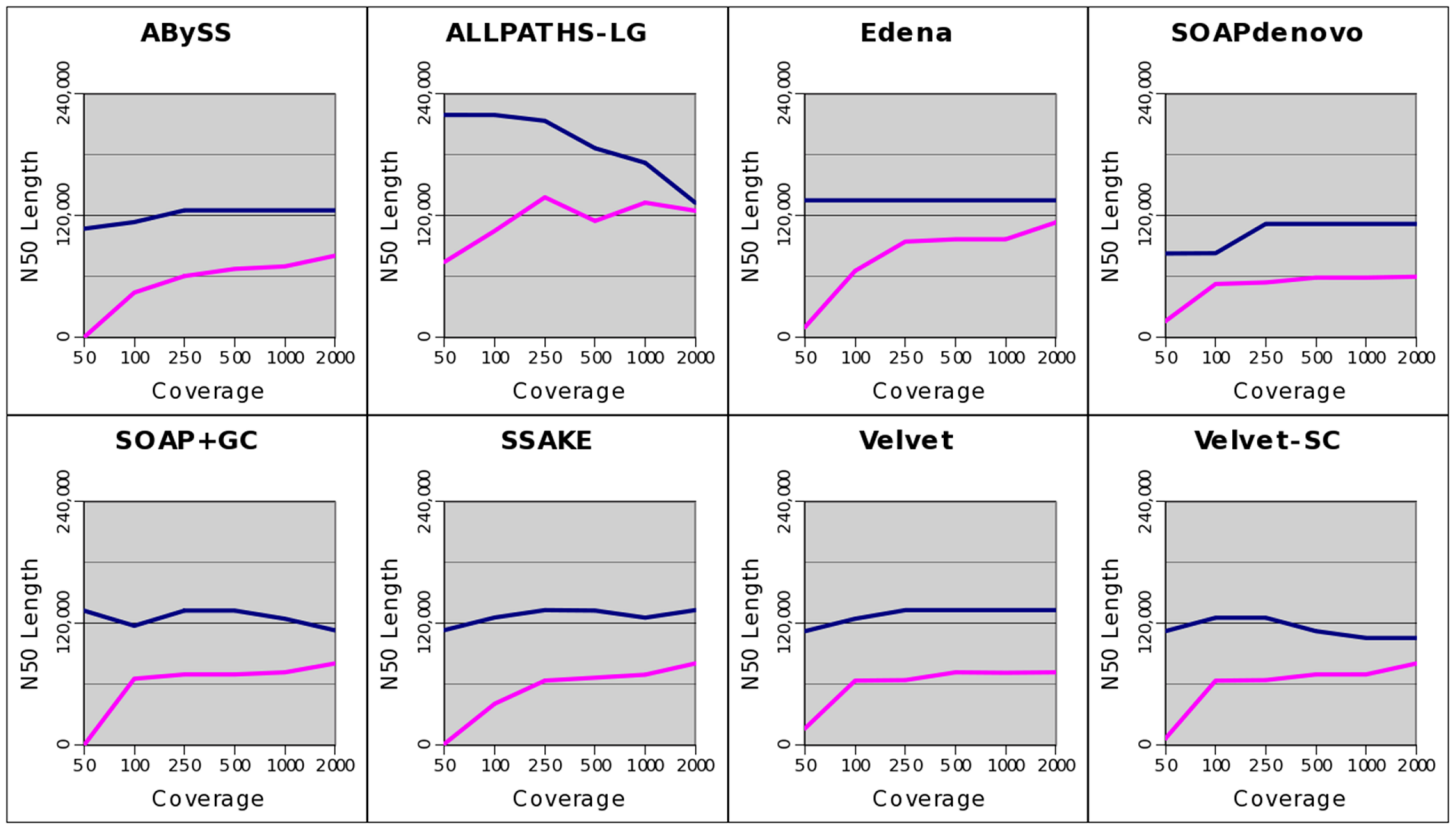
Completeness of the *E.coli* assemblies using data of various coverage. Assembly completeness is measured by N50 length of the corrected contigs, which are output by eight assemblers when treating simulated reads of various coverage (50X, 100X, 250X, 500X, 1000X, and 2000X) at a zero (blue line) and a strong positive GC bias (slope 3.6, pink line).

We found from Figure 5 that increasing coverage further indeed improved assembly contiguity for almost all assemblers. However, the improvements were much slower when coverage was increased from 250X to 2000X than from 50X to 100X. For SOAPdenovo and Velvet, no improvement could be observed when coverage was increased from 250X to 2000X. As a result, data of 2000X coverage still could not fully rescue the fragmentation of the *E. coli* assembly because of a strong positive GC bias. Nevertheless, the corrected N50 length of the Edena assembly at a strong positive GC bias was already quite close to that at no GC bias (84%) at 2000X coverage. Moreover, there were still signs of improvements if we kept increasing coverage for five assemblers. That is, it is possible to fully rescue the *E. coli* ’s assembly fragmentation due to GC bias if we increase the coverage even further. Note that we had also tried 5000X coverage, but some assemblies aborted without a clear reason. At 5000X coverage, the correct N50 length of the SOAP+GC assembly at a strong GC bias (65,689 bp) dropped lower than that at 2000X (80,518 bp). Thus, factors other than coverage may play a role at 5000X coverage. In any case, we show that the amount of data needed to rescue the same degree of GC bias is species dependent.

We also asked whether longer PE reads could rescue the *E. coli*‘s assembly fragmentation due to GC bias. We increased the length of simulated reads from 100 bp to 150 bp and repeated the assemblies at various data coverage (from 50X to 500X). Still, the more fragmented assembly of *E. coli* due to GC bias could not be rescued (Figure S5).

### Effects of GC Bias on Missing Assemblies of the Bacterial Genomes

In addition to N50 length, we examined the percentage of a reference genome that could not be aligned by any of the assembled contigs, which reflects the assembly missed by an assembler. For each bacterial genome, we checked the assemblies at three degrees of GC bias, at a strong negative, zero, and strong positive GC bias, using the eight assemblers. We took the assemblies obtained from the PE reads of 100X coverage and obtained the percentage of missing assembly from the GAGE outputs.

**Figure 6:**
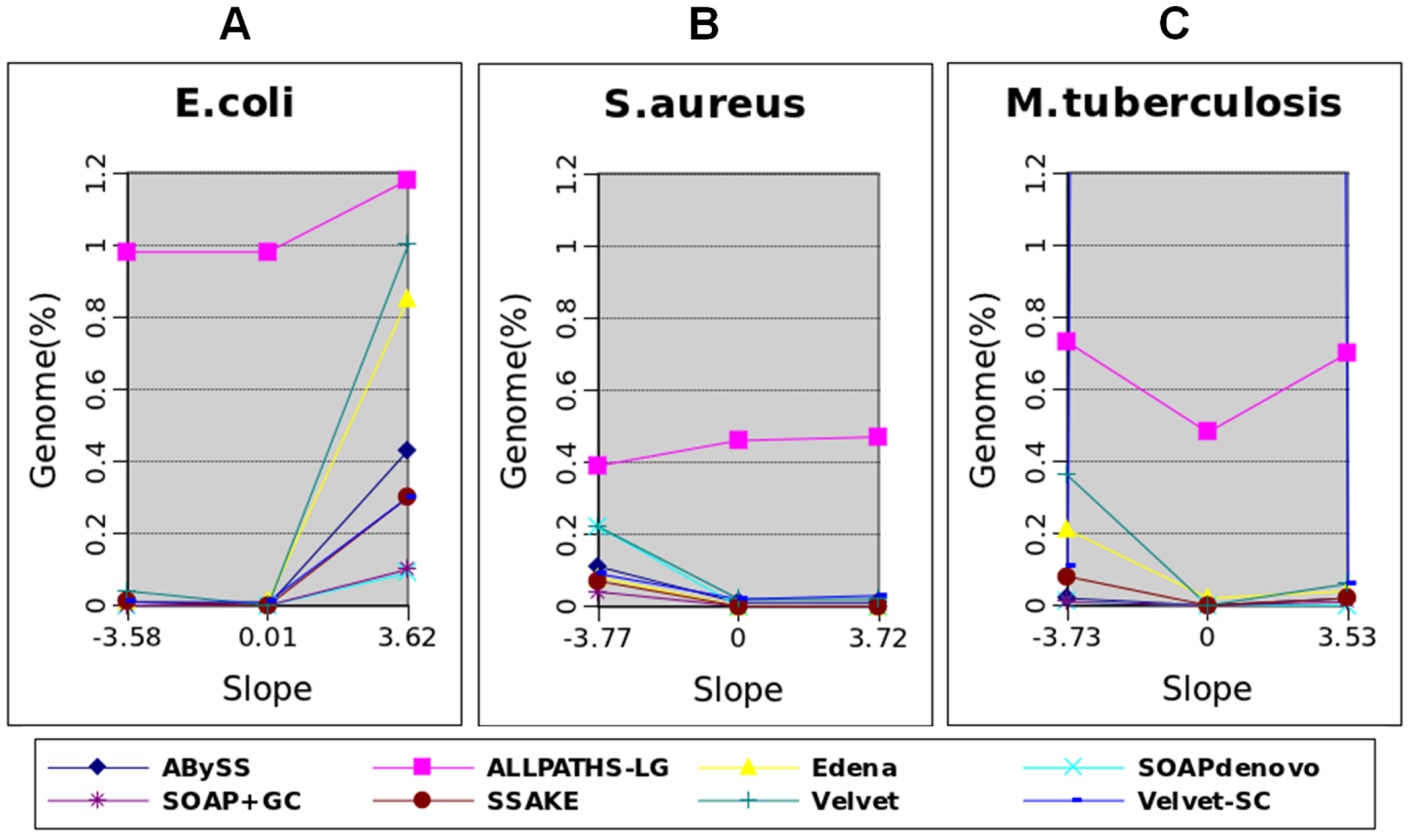
Percentage of unaligned reference sequences. The results are from the assemblies of three bacterial genomes: *E. coli* (A), *S. aureus* (B), and *M. tuberculosis* (C). Each of the eight assemblers treats data at a strong negative, zero, and strong positive GC bias.

Figure 6A shows the percentage of unaligned reference sequences at the three degrees of GC bias for all assemblers when treating the data of *E. coli*. For all assemblers, a strong positive GC bias resulted in a higher percentage of missing assembly than a zero GC bias did. In contrast, for all assemblers except Velvet, the percentages of unaligned reference sequences at a strong negative GC bias were about the same as those at no GC bias. This demonstrates the asymmetric effects of GC bias on missing assembly, i.e., the sign of GC bias matters.

We found that the effects of GC bias on missing assembly were also asymmetric in most of the *S. aureus* assemblies (Figure 6B). However, the asymmetry went in the opposite direction. For all assemblers except ALLPATHS-LG, the percentages of unaligned reference sequences were higher at a strong negative GC bias than those at no GC bias. At a strong positive GC bias, the percentages of missing assembly were about equal to those at no GC bias.

The effects of GC bias on missing assembly were relatively more symmetric in most of the *M. tuberculosis* assemblies. For all assemblers except Velvet-SC, the percentages of unaligned reference sequences at no GC bias were lower than those at either a strong negative or strong positive GC bias. In the ALLPATHS-LG, ABySS, and SOAPdenovo assemblies, the percentages of missing assembly were about the same at a strong negative and strong positive GC bias. For the remaining four assemblers, a strong negative GC bias resulted in a higher percentage of unaligned reference sequences than a strong positive GC bias did. Taken together, we found that a strong GC bias resulted in a higher percentage of missing assembly, which depends on the sign of GC bias and genomes.

### Effects of GC Bias on the Accuracy of Bacterial Genome Assemblies

Besides completeness, accuracy is an important quantity in assembly evaluation. We applied GAGE to capture various types of mis-assemblies, e.g., SNPs, INDELs, inversions, and translocations. The same assemblies of the three bacterial genomes in the last section were used for assessing accuracies at a strong negative, zero, and strong positive GC bias, respectively. We considered inversions, translocations, and INDELs of at least five bp as “major” errors, as GAGE breaks contigs at these loci. On the contrary, SNPs and INDELs of less than five bp were considered as “minor” errors.

In the case of *S. aureus* assemblies (Table 3), we observed a trend that a strong negative GC bias rendered more “major” errors than a zero and a strong positive GC bias did. This trend held for four assemblers, ALLPATH-LG, Velvet, Velvet-SC, and SOAP+GC. The trend was not clear in the ABySS, Edena, and SOAPdenovo assemblies because they made no “major” errors at all the three degrees of GC bias. SSAKE made five, four, and four “major” errors under a strong positive, zero, and strong negative GC bias, respectively. Similar to the effects of GC bias on missing assembly, the effects of GC bias on “major” errors were also asymmetric. That is, a strong negative GC bias resulted in more “major” errors than a strong positive GC bias did in general.

**Table 3:** Number of “major” errors in the assemblies at a strong negative, zero, and strong positive GC bias by the eight assemblers for the three bacteria.

Assembler	GC bias(slope)	S.aureus	E.coli	M.tuberculosis
ALLPATHS-LG	3.72	5	11	18
	0	5	15	22
	−3.77	6	14	27
ABySS	3.72	0	0	2
	0	0	0	1
	−3.77	0	0	3
Velvet	3.72	0	1	8
	0	1	0	12
	−3.77	2	5	14
Velvet-SC	3.72	11	14	21
	0	10	4	8
	−3.77	36	16	26
SOAPdenovo	3.72	0	0	0
	0	0	0	0
	−3.77	0	0	0
SOAP+GC	3.72	0	6	2
	0	1	4	1
	−3.77	2	3	2
Edena	3.72	0	0	0
	0	0	0	0
	−3.77	0	0	1
SSAKE	3.72	5	1	10
	0	4	0	11
	−3.77	4	1	6

For *E. coli*, we observed a trend that a strong negative and positive GC bias resulted in more “major” errors than a zero GC bias did (Table 3). This trend held for the four assemblers that made “major” errors in at least one degree of GC bias. In the case of *M. tuberculosis* assemblies, the relationships between GC bias and “major” errors were not consistent among all assemblers (Table 3). We repeated the above analyses for “minor” errors, but found no trend in the assemblies of the three bacterial genomes (Table S1, Table S2 and Table S3).

### Relationship between Mis-assembly and Coverage

Here, we explored the mechanisms behind the effects of GC bias on the mis-assembly of bacterial genomes. Specifically, we studied the relationship between mis-assembly and read coverage. For each bacterial genome assembly, we collected the assembly errors reported by GAGE, including unaligned reference sequences and assembly errors, and denoted them on the genome. The read coverage could be readily obtained since we used simulated data. We checked whether the read coverage at the erroneous loci was aberrant.

**Figure 7:**
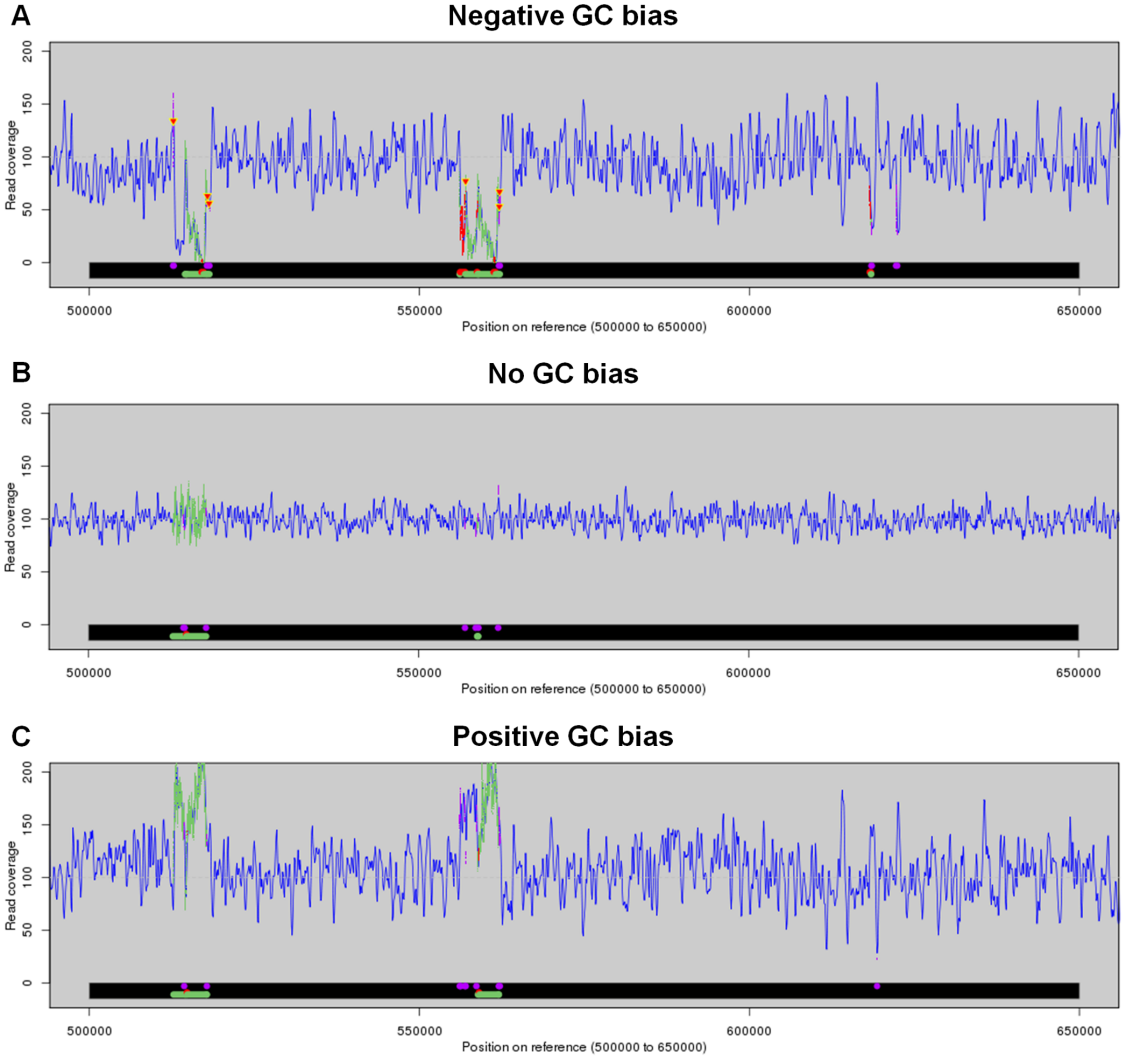
Read coverage and mis-assemblies on the *S.aureus* genome. Read coverage (blue curves) and mis-assemblies (colored regions in the bottom bar) in a region of *S. aureus* genome at a strong negative (A), zero (B), and strong positive (C) GC bias. Different colors represent different types of mis-assemblies: tandem repeat error (green), translocation error (purple), unaligned reference sequence (red). The colors are projected to the curve of read coverage. The down-triangles in the coverage curves denote single-base insertions.

Figure 7 shows the read coverage in a region of the *S. aureus* genome, resulted from the data at a strong negative, zero, and strong positive GC bias, respectively. Compared with the case at no GC bias, both negative and positive GC biases led to a larger area of tandem repeat errors (green color, around 560 kb) and more unaligned regions (red in the bottom bar, around 560 kb). The corresponding read coverage at the error loci was clearly lower and higher than the mean value at a negative and positive GC bias, respectively. Around the 620 kb region, positive and negative GC bias introduced one and two translocation errors respectively, while negative GC bias further led to an tandem repeat error and an unaligned region. The read coverage at these error loci was lower than the mean value in both cases at a negative and positive GC bias. Note that aberrant coverage did not always correlate with mis-assembly. Around the 520 kb region, the same types of errors existed even in the case at no GC bias.

**Figure 8:**
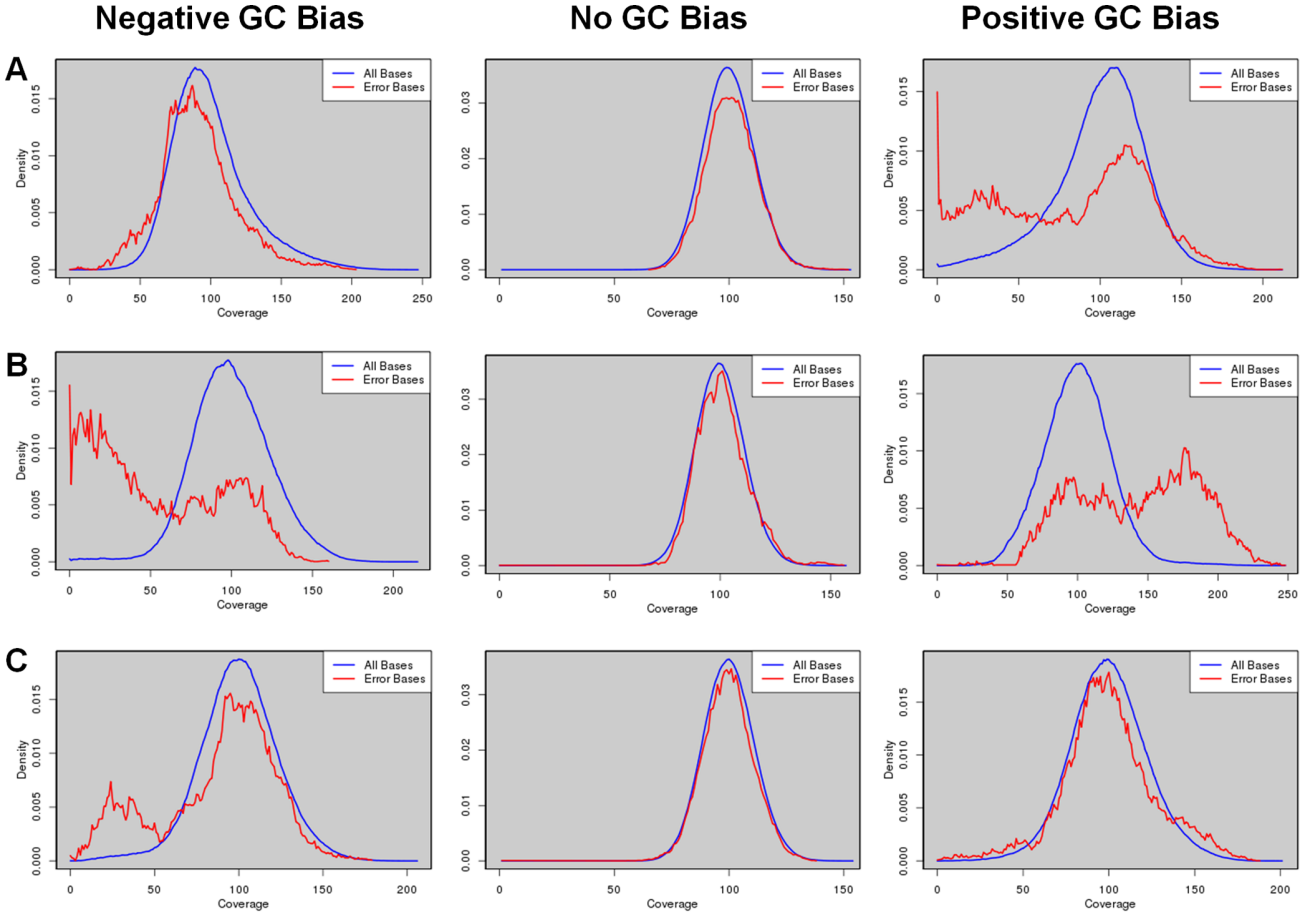
Distributions of coverage depths at all bases and at error bases. Distributions of coverage depths at error bases (red curves) are compared with those at all bases (blue curves) in the Velvet assemblies of three bacterial genomes: *E. coli* (A), *S. aureus* (B), and *M. tuberculosis* (C), using data simulated at a strong negative (left column), zero (middle column), and strong positive (right column) GC bias.

To explore the overall relationship between mis-assembly and read coverage, we plot the distribution of coverage depths at all genomic loci where errors occurred, which was then compared with the background distribution, i.e., the distribution of coverage depths across the whole genome. The results of the *E. coli* assemblies by Velvet were shown in Figure 8A. We found that at a strong negative GC bias, the distribution of coverage depths at error loci was shifted toward a lower coverage compared with the background distribution. Such a shift in distribution disappeared in the case of no GC bias. At a strong positive GC bias, we observed a clear enrichment of low coverage depths at the error loci, as well as a slight enrichment of high coverage depths. The distributions of coverage depths at a strong negative and positive GC bias differed from the background in a similar fashion for all other seven assemblers (Figure S6). This suggests that the mechanisms behind the additional errors because of GC bias were similar for all the assemblers under study. Moreover, these additional errors could be explained by the aberrant coverage at the corresponding loci.

We repeated the above analyses for the assemblies of two other bacterial genomes by all eight assemblers, and the results by Velvet were shown in Figure 8. At a strong negative GC bias, we observed a clear enrichment of low coverage depths at the error loci in the assemblies of both *S. aureus* (Figure 8B) and *M. tuberculosis* (Figure 8C). At no GC bias, the distributions of coverage depths at the error loci again followed closely the background distributions. At a strong positive GC bias, we observed a clear and a slight enrichment of high coverage depths at error loci in the assemblies of the *S. aureus* and *M. tuberculosis* genomes, respectively. On the other hand, we observed only a tiny and a slight enrichment of low coverage depths at the loci of error in the assemblies of the *S. aureus* and *M. tuberculosis* genomes, respectively. Again, the enrichments of low and high coverage depths at error loci were similar in the results of all other seven assemblers (Figure S6). Taken together, based on the enrichments of low or high coverage of reads at error loci because of GC bias for all the three bacteria, aberrant coverage can explain the assembly errors due to GC bias.

In the assemblies of the three bacteria, we observed different extents of enrichment of low or high coverage at a strong GC bias, which suggests distinct natures of errors in the three cases. Indeed, we found a perfect correlation between the clear enrichment of low coverage depths at strong GC bias and the percentage of missing assembly. In the *E. coli* assemblies, low coverage depths were clearly enriched at a strong positive GC bias, but not enriched at a strong negative GC bias (Figure 8). Consistently, the percentage of unaligned reference sequences errors was much higher at a strong positive GC bias, but not higher at a strong negative GC bias (Figure 6). In the *S. aureus* and *M. tuberculosis* assemblies, low coverage depths were clearly enriched at a strong negative GC bias, but not enriched at a strong positive GC bias. Again, we observed higher percentages of unaligned reference sequences at a strong negative GC bias, which were not higher at a strong positive GC bias. Thus, a clear enrichment of low coverage depths can explain missing assembly.

To further explore the natures of assembly errors due to GC bias, we decomposed the errors into nine categories and plot again the distributions of coverage depths at error loci for each category. Figure S7 showed the distributions of coverage depths of three major types of errors: missing assembly, tandem repeat errors, and mis-joins in the Velvet assemblies of the three bacterial species. Indeed, in the three cases of clear enrichments of low coverage depths, clear missing assemblies were observed. In addition, clear enrichment of low coverage could explain other types of errors. For example, at a strong positive GC bias, mis-join errors constituted the majority of the enriched low coverage depths in the Velvet assembly of *E. coli* (Figure S7). In the case of *S. aureus*, tandem repeats contributed to most of the enrichments of low and high coverage depths.

### Effects of GC Bias on the Assemblies of more Complex Genomes

To explore the effects of GC bias on the assemblies of more complex genomes, we took for analyses chromosome one and five of two plants, *A. thaliana* and *O. sativa*, which lengths were 30.4 Mb and 30.0 Mb, respectively (Table 2). From each chromosome, we simulated PE reads of length 100 bp at three degrees of GC bias (slope −3.8, 0, and 3.7), each to 100X coverage. Again, we assembled these data sets using the eight assemblers, applied GAGE to detect mis-assembly, and obtained assembly statistics.

**Figure 9:**
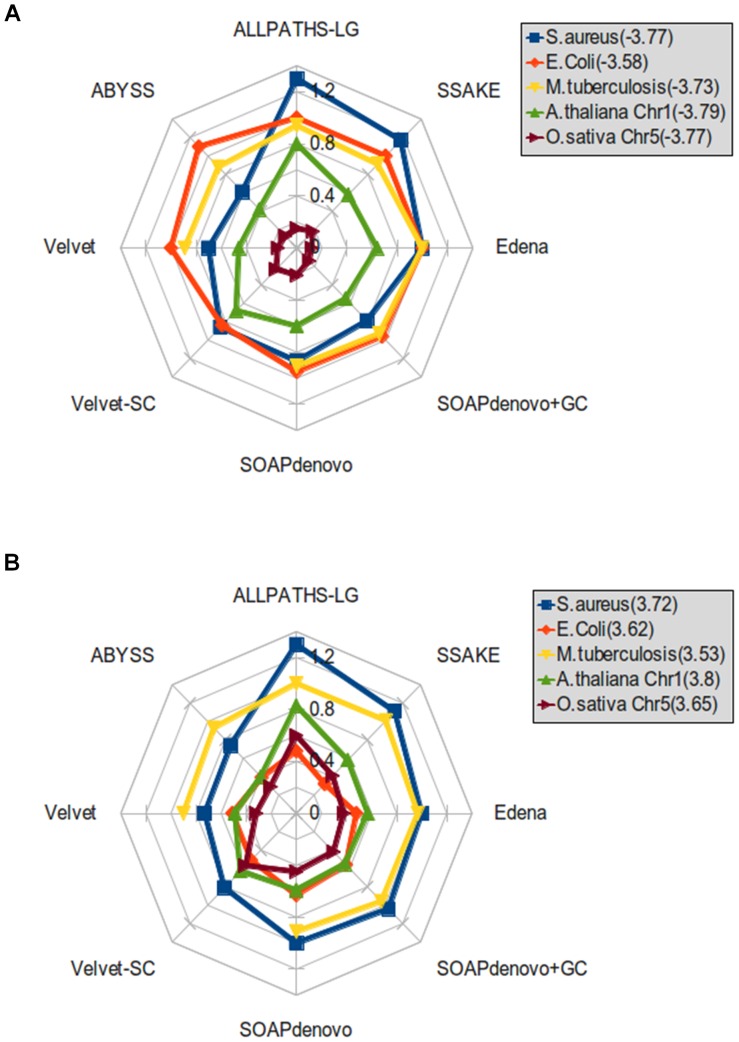
Ratio of corrected N50 length at a strong GC bias to that at no GC bias. Ratio of the corrected N50 length at a strong negative GC bias (A) and a strong positive GC bias (B) to that at no GC bias when assembling the data of five species (in different colors) using eight assemblers.

We found that the effects of GC bias on the completeness of assembly were greater in general for the two plant chromosomes than for the three bacterial genomes. For all assemblers, we calculated the ratio of the corrected N50 length at a strong negative or positive GC bias to that at no GC bias for the three bacterial genomes and the two plant chromosomes. A smaller ratio indicates stronger effects of GC bias on assembly completeness. At a strong negative GC bias, the corrected N50 length dropped the most in the *O. sativa* assemblies by all assemblers (Figure 9A). The ratios in corrected N50 length were the second smallest in the *A. thaliana* assemblies by all assemblers. At a strong positive GC bias, the ratios in corrected N50 length stayed the lowest in the *O. sativa* assemblies by five of the eight assemblers (Figure 9B). The second lowest ratios were from the *E. coli* assemblies while the ratios from the *A. thaliana* assemblies ranked third. Note that among the five species, the variation of GC contents in the *O. sativa* genome is also the greatest (Table 2). The standard deviation of GC contents in the *A. thaliana* and *E. coli* genomes are the second and third largest, respectively. These suggest a relationship between variation of GC contents and the effects of GC bias on the completeness of assembly.

For the two plant chromosomes, we observed clear effects of GC bias on the percentage of missing assembly (Figure S8). In the results of all assemblers except the *A. thaliana* assembly by ALLPATHS-LG, strong positive and negative GC bias resulted in a higher percentage of unaligned reference sequences. Compared with the results of the bacterial genome assemblies, the percentages of missing assembly were greater in the two plant cases.

As for the accuracy of assembly, the numbers of “major” errors in the two plant assemblies at a strong negative and positive GC bias were greater than those at no GC bias for all assemblers except SSAKE (Table S4 and Table S5). When checking “minor” errors in the *A. thaliana* assemblies, we found that a strong negative and positive GC bias resulted in more “minor” errors for all assemblers expect Velvet-SC (Table S4 and Table S5). In the *O. sativa* assemblies, however, the effects of GC bias on “minor” errors were obscure, i.e., observable in only four out of the eight assemblers.

Taken together, we observed similar trends in the effects of GC bias on the assemblies of the two plant chromosomes as of the three bacterial genomes. The impacts tend to be greater in the assemblies of the two more complex genomes, which correlates with the larger variations of GC contents of their genomes.

### Distribution of GC Contents and Effects of GC Bias

**Figure 10:**
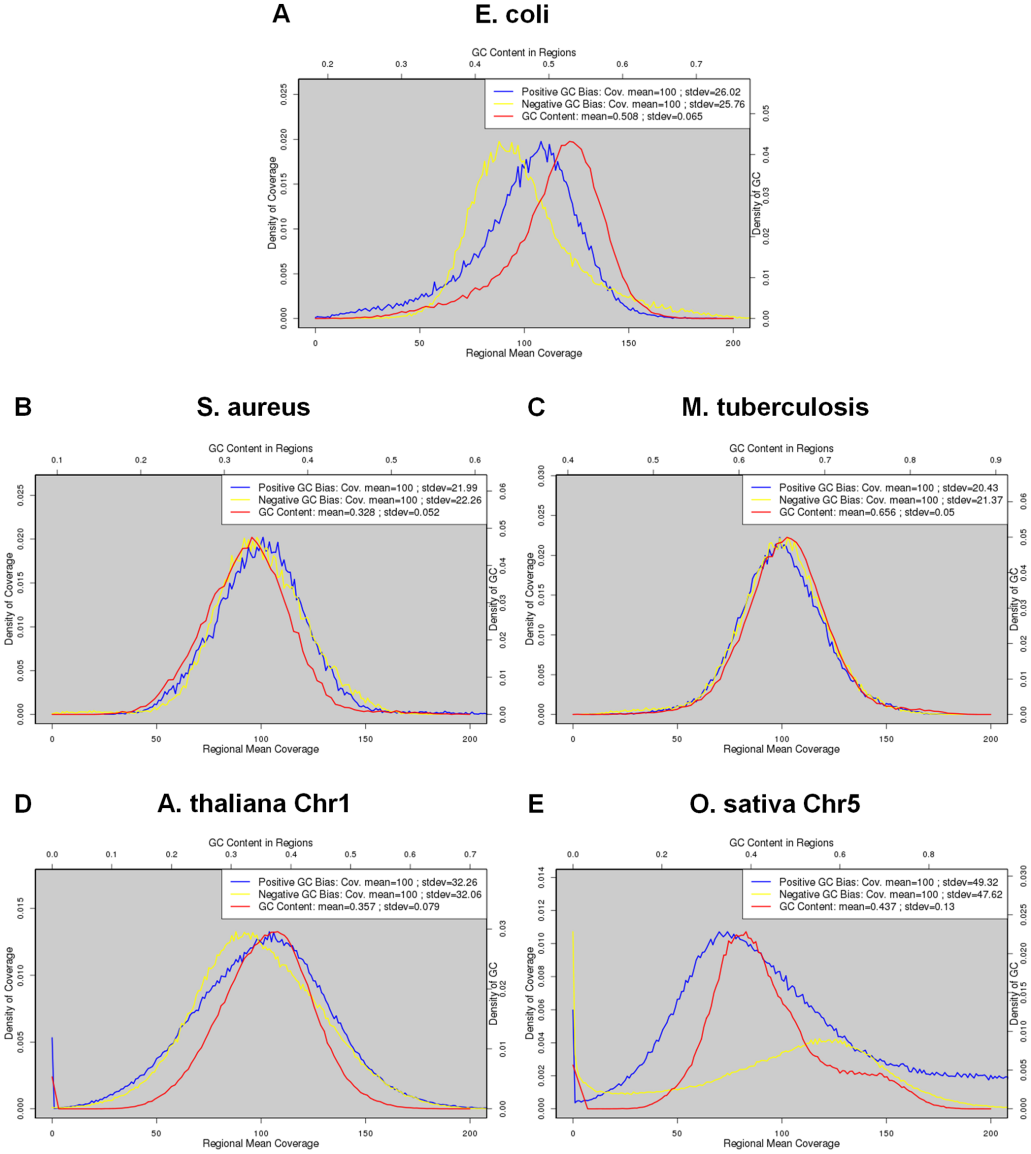
Distribution of GC contents and read coverage of the five species under study. The red curves stand for GC contents (scale in top axis). The blue and yellow curves represent read coverage at a strong positive and strong negative GC bias, respectively (scale in bottom axis). We used the data at 100X coverage for the five species.

We had shown a correlation between read coverage and the effects of GC bias. Besides, the variation of GC contents was found related to the effects of GC bias. Here, we further explored how the variation of GC contents posed an effect through read coverage. For all species, we plot the distributions of GC contents as well as the distribution of read coverage across the genome (Figure 10).

The distribution of GC contents of the *E. coli* genome was not symmetric (Figure 10A). The frequency dropped more slowly to zero as GC content decreased than as it increased. Thus, there were still genomic regions of extremely low GC contents. At a strong positive GC bias, this resulted in many regions of extremely low coverage. On the contrary, there was almost no region of extremely low coverage at a strong negative GC bias. This likely explained the fact that at 100X coverage, the *E. coli*‘s assembly fragmentation due to a strong negative GC bias could be rescued, but could not be rescued when the GC bias was strongly positive. For the two other bacteria, there were almost no regions of extremely low coverage at either a strong positive or strong negative GC bias (Figure 10B and 10C). Consistently, the assembly fragmentations due to GC bias could be rescued at either sign of bias. For *O. sativa*, the distribution of GC contents skewed toward high GC (Figure 10E). Thus, there were many more regions of extremely low coverage at a strong negative GC bias than at a strong positive GC bias. Consistently, we found stronger effects of negative GC bias on assembly completeness (Figure 9).

The effects of GC bias on missing assemblies also made sense in the light of the distribution of GC contents. For *E. coli* and *O. sativa*, missing assemblies were more serious at a strong positive and strong negative GC bias, respectively (Figure 6 and Figure S8). For the rest three species, the effects of GC bias on missing assembly were more symmetric. Note that in the *S. aureus* case, we still observed a weak asymmetry in the percentage of missing assemblies, which could not be explained by its distribution of GC contents.

The distribution of GC content could also explain the slow rescue of the *E. coli* ‘s assembly fragmentation due to a strong positive GC bias as we increased read coverage. That is, the read coverage at regions of extremely low GC contents increased very slowly. To support this argument, we counted the number of genomic regions of zero coverage. At read coverage of 50X, 100X, 250X, 500X, 1000X, and 2000X, the numbers were 57, 29, 20, 17, 13, and 11, respectively. The total number of bases in these regions were 3618, 2214, 1022, 745, 539, and 374, respectively. Thus, there still exist regions of zero coverage even at 2000X read coverage.

### Fluctuations of Read Coverage in Simulation

**Figure 11:**
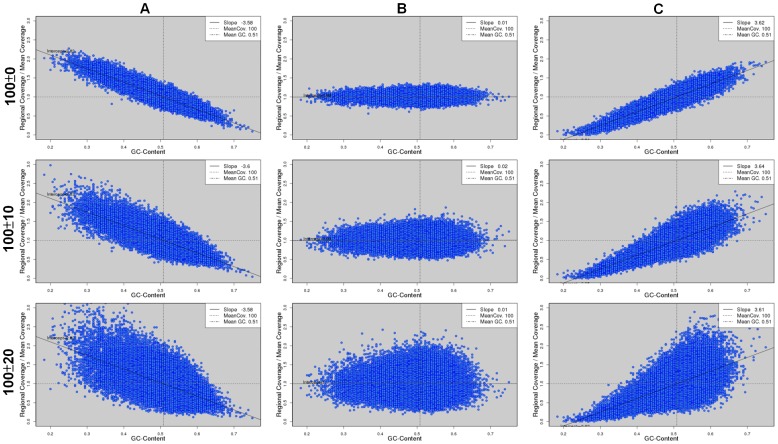
Scatter plots of GC content and read coverage of data simulated with various degrees of background fluctuations. The data are simulated from the *E. coli* genome at three degrees of background fluctuations: zero (top row), 10 (middle row), and 20 (bottom row). At each degree of background fluctuation, we simulated PE reads at a strong negative (A), zero (B), and a strong positive (C) GC bias, respectively.

In addition to the degree of GC bias, a simulation can be controlled by other factors. For example, there can be genuine fluctuations in coverage depths not because of GC bias. Here, we explored the impact of this factor on the assemblies of the three bacterial genomes. During read simulation, we introduced a background fluctuation in read coverage across the genome before considering GC bias. Note that the background fluctuation was introduced independently from GC content. Again, we simulated PE reads of length 100 bp to a 100X coverage at a strong negative, zero, and strong positive GC bias, respectively. The background fluctuation was controlled by a standard deviation and we simulated data at two values of standard deviation (10 and 20). We observed clearly a wider range of read coverage as the background fluctuation increased (Figure 11), which covered the possible ranges of read coverage in real Illumina data (Figure S1).

**Figure 12:**
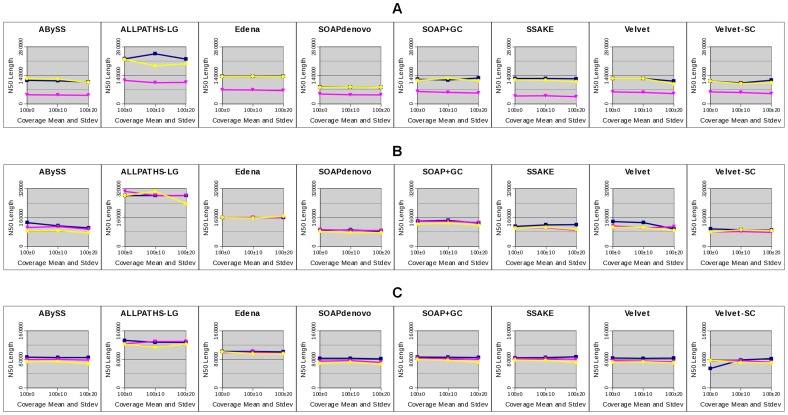
Corrected N50 length of assemblies at three background fluctuations. We show the corrected N50 length in eight assemblies of three bacterial genomes: *E. coli* (A), *S. aureus* (B), and *M. tuberculosis* (C), using simulated data at three degrees of background fluctuations (x-axis), each at three degrees of GC bias: negative (yellow), zero (dark blue), and positive (pink).

Among all the assemblies, we only observed a trend that a greater fluctuation in read coverage led to a slightly lower corrected N50 length in the *E. coli* assemblies at a strong positive GC bias by all eight assemblers (Figure 12). The fact that no other consistent trend could be observed indicates that background fluctuation did not affect genome assembly in general. When they indeed play a role, the effect is only minor.

### Estimation of GC Bias in Real NGS Data without a Reference Genome

For our study to be helpful for *de novo* genome assembly, a prior knowledge of the degree of GC bias in the real data is necessary. However, estimating the degree of GC bias is difficult without a reference genome. Here, we provided an alternative approach of estimating the degree of GC bias in the real data without a reference genome. The idea is to replace the reference genome with the contigs assembled from the data.

**Figure 13:**
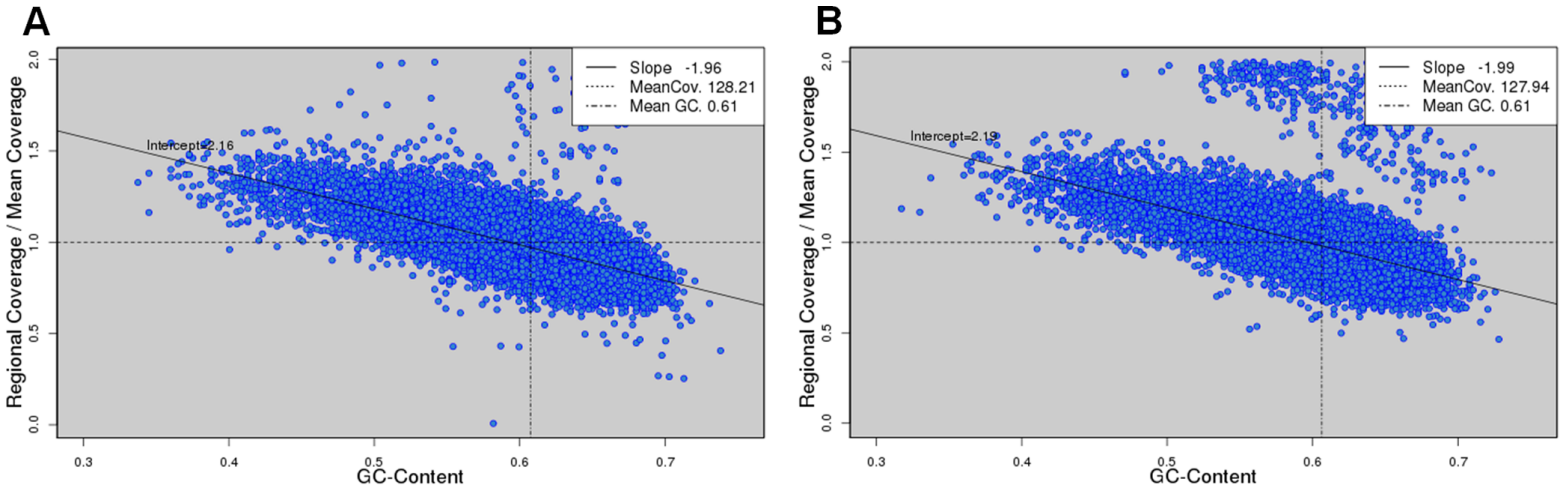
Estimation of the degree of GC bias using reference sequences and assembled contigs. We show the scatter plots of GC content and read coverage for *P.fluorescens* Pf0-1 Illumina library (DRR001171) based on the known reference genome (A) and the contigs assembled by Edena, which contain 6,610,650 bases and the N50 length is 8,257 (B).

We assembled the real Illumina reads using Edena, Velvet, and ABySS. The Edena assembly of the DRR001171 library contained 6,610,650 bp in total, which was close to the size of the *P. fluorescens* Pf0-1 genome, 6.44 Mb. The N50 length of this assembly was 8,257 bp. Using these contigs as reference sequences, we calculated GC content and read coverage across the genome. The resulting scatter plot was rather similar to that when a true reference genome is used (Figure 13). The estimated degree of GC bias, −1.99, was also close to the value when a true reference genome was used (−1.96).

**Figure 14:**
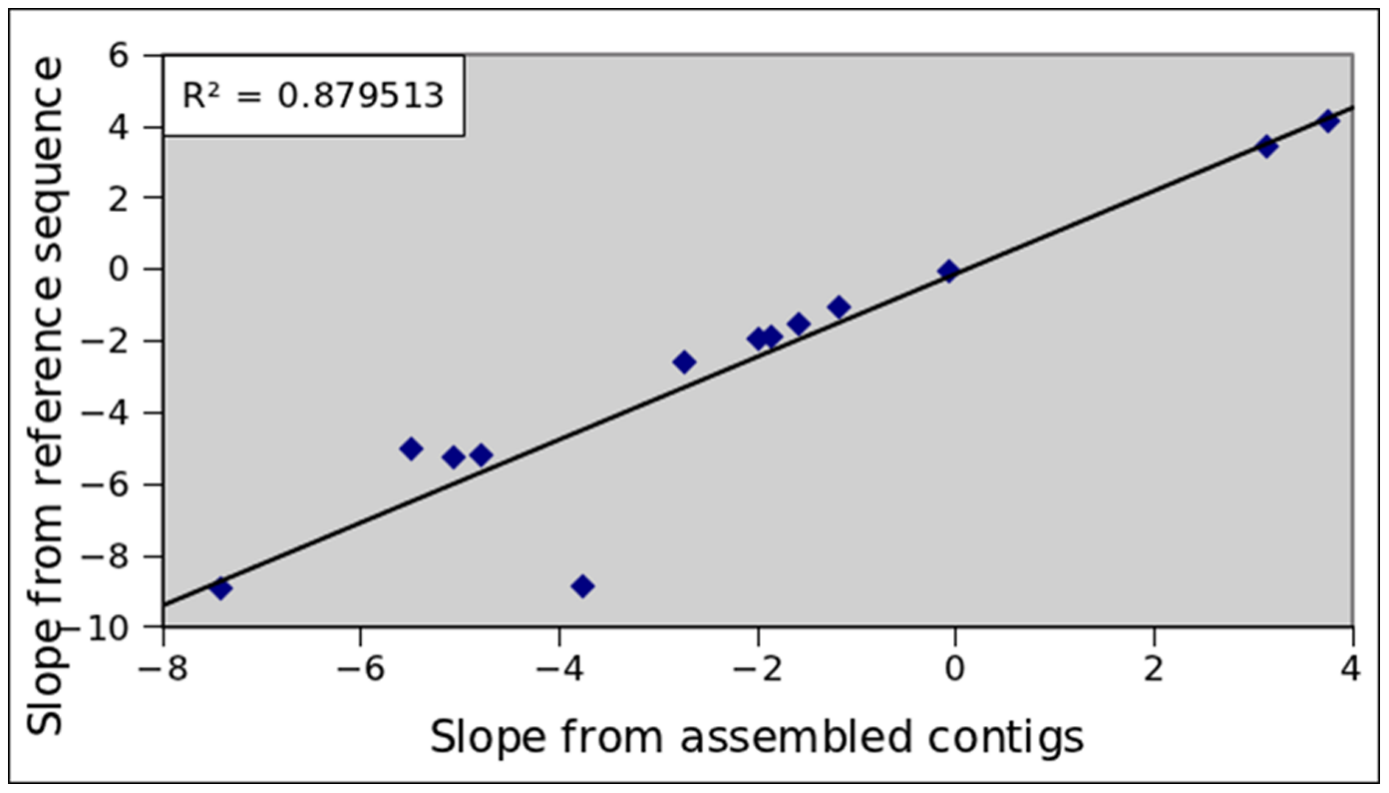
Correlation between the degree of GC bias obtained using reference sequences and assembled contigs. The correlation is calculated for thirteen Illumina data sets, including eight data sets by Edena, four data sets by Vevlet and one data set by ABySS. The high R^2^ value (0.88) indicates that estimating the degree of GC bias using the assembled contigs is appropriate.

To prove the generality of this approach, we repeated the above estimations for all the Illumina data sets (Figure S9), excepting SRR022867 because the assembly aborted. Figure 14 shows the relationship between the degree of GC bias estimated using a reference genome and that using the assembled contigs. We observed a strong correlation (R^2^ = 0.88) between the two values. Thus, the degree of GC bias can be estimated rather accurately in this approach.

## Discussion

### Trend of GC Bias

In this study, we captured GC bias by a linear relationship between GC content and read coverage. A linear relationship between GC content and read coverage have been reported in previous studies [12]. Benjamini et al. recently claimed a uni-modal GC bias, i.e., both GC-rich and GC-poor regions are under-represented in read coverage [34]. The authors thought that linear relationships observed in previous studies were the results of only capturing the GC-poor parts because the genomes are GC-poor. We did find a uni-modal GC bias in one of our real data sets, SRR001666 of *E. coli* (Figure S1). However, we did not observe a uni-modal GC bias in the three other data sets of *E. coli*, in which only a linear relationship could be observed (Figure S1). Moreover, in the three data sets of *S. aureus*, which genome is GC-poor and the majority of GC contents across the genome are below 50%, we observed an increase in read coverage when the GC contents drop to the extreme. This is exactly opposite to the behavior of a uni-modal GC bias. These indicate that the behavior of GC bias is greatly determined by experimental procedures, which varies between laboratories and from run to run. As most of our data sets presented a linear relationship between GC content and read coverage, we decided to capture the behavior of GC bias using a linear model. A linear model is also fundamental in a sense that uni-modal GC bias can be described by the combination of two linear models. Thus, one can obtain some insights into the results of uni-modal GC bias from our observations made on linear GC bias.

In twelve of the fourteen libraries, the GC bias is negative (Table 1). This is consistent with a previous report that the amplification of GC-rich regions is less efficient [35]. Some methods have been developed to increase the amplification efficacy of the GC-rich regions [36], [37], [38]. However, we cannot find evidence that the two libraries with a positive GC-bias were prepared with these methods. Interestingly, the GC-biases in three *S. aureus* libraries are stronger than those in other libraries (Table 1) and they are prepared with a different polymerase and PCR cycles. It has been reported that more PCR cycles increase GC bias [39]. But the sample number is too small to conclude this observation.

### Definition of the Degree of GC Bias

As we explored the linear relationship between GC content and read coverage, it is natural to define the degree of GC bias as the slope of the line fitting the data points. This slope quantifies the tendency of a higher (or lower) read coverage in a genomic region of a higher GC content. Another possible measure of the relationship between GC content and read coverage is correlation, which is not equivalent to slope. Slope should be a better measure of the degree of GC bias than correlation for two reasons. First, correlation is inert to scaling, i.e., when x- or y-axis is rescaled by a factor, the resulting correlation remains the same. In a scatter plot of a strong GC bias, if we reduce the deviations of read coverage from the mean value by a large factor so that all the points stay very close to the mean coverage, a strong GC bias cannot be concluded anymore. However, the correlation after reducing the deviations remains equal to the original value at a strong GC bias. Note that reducing deviations is different from normalizing the coverage to the mean value. Secondly, at a fix slope, correlation can be affected by the fluctuations in read coverage. When we introduced background fluctuations in read coverage, the ranges of read became wider (Figure 11), leading to a smaller correlation. However, we found that background fluctuation did not affect genome assembly at strong GC bias in general. This fact cannot be reflected by correlation.

### Variation of GC Contents

In this work, we found that the degree of GC bias did not correlate with the mean GC content or with the variations of GC contents across the genomes. These indicate that genomic content itself is not sufficient for generating GC bias. However, we emphasize that the variation of GC contents is a necessary component of GC bias. If GC contents remain identical throughout a genome, we expect no GC bias at all no matter what kind of library preparations and PCR amplification protocols are applied. In fact, it is impossible to define GC bias when there is no variation of GC contents across a genome.

### Illumina PE Read Simulation

It is advantageous to use simulated reads for studying the effects of GC bias on genome assembly. First, one can tease apart GC bias from other various types of sequencing artifacts. Sequencing artifacts in real data arise from various sources, e.g., reads from non-desired genomes due to sample contamination [40], non-genuine reads of low complexity because of insensitive fluorescence detection [41], chimeric reads (i.e., sequences joined from two genomic loci) that occur in the sample manipulation step [11], …, etc. If real data is used for studying the effects of GC bias, it will be difficult to discern whether the effects are indeed from GC bias rather than from other sequencing artifacts. Note that, however, our simulation still took into account common sequencing errors (Methods). Secondly, via simulation we can control the degree of GC bias. Although experimental protocols have been proposed to reduce GC bias in real data [13], [15], it is not clear how to control the degree of GC bias experimentally. Without covering a full range of degrees of GC bias, it is difficult to judge at which point GC bias starts to pose an impact on genome assembly. Finally, economical data source is also of a great concern.

### Optimization of Assembly

In this work, assemblies were optimized to render the largest N50 length. This approach emphasizes on assembly contiguity but not quality. Our results thus applied only within this scope. It is possible that an assembly with a smaller N50 length contains fewer errors and its corrected N50 length turns out larger or other statistics turned out better. This strategy, however, is commonly used because longer contigs are usually preferred [32].

### Effects of GC Bias, Read Coverage, and Distribution of GC Contents

Several of our results point to the argument that low coverage of reads due to GC bias explains the major effects of GC bias on the completeness and accuracy of genome assembly. First, we found that the assembly fragmentation due to GC bias could be rescued via supplying more data. These extra data increase the read coverage in the genomic regions of a relatively low or high GC content when the GC bias is positive or negative, respectively. Secondly, we observed a perfect correlation between the clear enrichment of low coverage depths at a strong GC bias and the percentage of missing assembly. Thirdly, in all the assemblies of three bacterial genomes at a strong negative or positive GC bias, we observed various degrees of enrichments of low coverage depths at error loci compared with the case at no GC bias. Fourth, the reduction in assembly contiguity correlates well with the abundance of low coverage regions. The more low coverage regions, the corrected N50 length drops more.

High coverage due to GC bias also poses an effect. In the assemblies of the *S. aureus* genome, we observed a clear enrichment in high coverage at error bases. At high coverage, the errors were mostly tandem repeat errors. We guess it related to the fact that in many assemblers repeats are characterized based on coverage.

Aberrant coverage due to GC bias is clearly induced by the distribution of GC contents. We reveal that the shape of GC content distribution governs the distribution of read coverage, which in turns affects genome assembly in many respects. For example, the distribution of GC contents of the *E. coli* genome is not symmetric and enriched in low GC contents. Consistently, the assembly fragmentation due to a strong negative GC bias can be fully rescued by doubling the data, but the rescue is only partial when GC bias is strongly positive. The asymmetric distribution of GC contents also explains the asymmetry of missing assemblies in many cases. Note that not every effects of GC bias can be explained by the distribution of GC bias. For example, the distributions of GC contents of *S. aurus* and *M. tubeculosis* are rather symmetric. However, the distributions of read coverage at error bases are not symmetric at all. This suggests more subtle mechanisms behind.

When Illumina data is GC biased, low read coverage is the results of several factors. These include the degree of GC bias, overall amount of data, and the variation of GC contents. These factors likely couple together. For example, in the assemblies of the three bacterial genomes, we found that a weak GC bias (slope between −2 and 2) did not affect assembly completeness. The threshold is likely smaller in the case of *O. sativa* assemblies because the variation of its GC contents is greater so that the same ratio of low coverage regions can be achieved with a smaller slope.

### Rescuing Assembly Fragmentation due to GC Bias

We have shown that in principle assembly fragmentation due to GC bias can be rescued with more data. However, the amount of data needed for a full rescue depends closely on the distribution of GC contents. This can be challenging in practice if the required coverage is very high. First, even for a small genome, assembling a very large amount of data is time-consuming and memory intensive. Second, assembly may not be improved with a large amount of real data. Real data usually contain errors of various natures. These errors may deteriorate assembly when they accumulate. Even using our simulated reads, we observe a clear drop in assembly contiguity when we assembled *E. coli* ‘s data of 2000X using ALLPATHS-LG and of 5000X using SOAP+GC. Lastly, the increasing sequencing cost for more data is less affordable. These practical challenges make it even more difficult to fully rescue assembly fragmentation due to GC bias for large genomes. Nevertheless, we emphasize again that the effects of GC bias are contributed by several factors. If the GC contents do not vary a lot or the degree of GC bias is small, it is still possible to fully rescue assembly fragmentation due to GC bias. If a full rescue is not possible, at least a significant partial rescue can be achieved by doubling the coverage because the rescue is most efficient at this coverage range.

### Repeat and GC Bias

Read coverage is a useful piece of information adopted in many assemblers. For example, Velvet decides whether an assembled contig is “unique” or “repetitive” in the genome based on read coverage, and performs repeat resolution starting from the “unique” contigs. Uneven coverage thus can lead to in-correct judgments of contig uniqueness by assemblers. For example, at a positive GC bias, a unique contig of a high GC content may be considered as “repetitive” because of its high coverage of reads. Thus, high coverage may also be harmful to assembly. This statement is supported by our observation of a clear enrichment of high coverage depths at error loci in the *S. aureus* assemblies at a strong positive GC bias.

It is interesting to discuss the effects of GC bias when repeats locate within a GC-poor or GC-rich region. We first explored whether repeats locate mostly in the GC-poor or GC-rich regions of our five genomes under study. We used PILER [23] to identify repeats in each genome and plot the distribution of GC contents in repeats (Figure S10). For *S. aureus*, there was indeed a significant portion of repeats in GC-rich regions. Such a feature was much weaker for the rest four species. For *E. coli* and *M. tuberculosis*, there were only slight enrichments in high GC contents within repeats. Combined with the results above, the proportion of repeats in GC-rich regions does not correlate with the effects of GC bias on assembly completeness. For example, at 100X read coverage, we observed no effects of GC bias on the completeness of the *S. aureus* assembly.

We hypothesize that repeats within GC-poor or GC-rich regions should not affect assembly much. This issue is a bit complicated because it couples with read coverage. At a very strong positive (or negative) GC bias, read coverage in a GC-poor (or GC-rich) region drops to very low or even zero. This clearly disrupts assembly and should be irrelevant to GC content. When GC bias is not that strong, the read coverage in repeats is only reduced or enhanced, but not depleted. We then need to consider how repeats are resolved. In principles, a repeat longer than read length or insert size is not resolvable. Thus, a non-resolvable repeat should remain non-resolvable whether the repeat is in a GC-poor/rich region or not. For a resolvable repeat, it then depends on how each assembler resolves the repeat. For Velvet, we guess that read coverage in regions flanking a repeat is more important than read coverage at the repeat.

In addition to these discussions, we quantitatively compared the effects of repeats and GC bias on genome assembly as follows. For each of the three bacterial genomes, we detected repeats using PILER and calculated GC contents using a sliding window of size equal to the mean fragment length (Methods). We broke the genome into pieces by removing the repeats, and indicated the effect of repeats by the N50 length of the resulting sequences. To assess the effect of GC bias, we broke the genome into pieces by removing the windows with an extreme GC content. Specifically, we picked the top 25 windows with the highest (or lowest) GC content for breaking the genome and calculated the resulting N50 length. We increased the number of windows to 500 with a step size 25 and repeated the procedure. We plotted the N50 lengths in cases of repeats and various degrees of GC bias for comparing their effects on assembly contiguity (Figure S11).

In principle, the effect of GC bias should appear on top of the effect of repeats because it is not possible to leave out the effects of repeats during assembly. However, we first considered GC bias alone without removing repeats from genomes for a “clean” comparison. In Figure S11, the N50 length at a relatively small GC bias, i.e., when the number of extremely high or low GC windows being removed was small, was longer than the N50 length in case of repeats. When GC bias got stronger, the N50 length under GC bias dropped lower than the N50 length in case of repeats for all three genomes. These observations indicate that the “shear” effect of GC bias can be greater than the effect of repeats when the GC bias is relatively strong. When repeats are always removed, quantifying the effects of repeats and GC bias is arbitrary. If we quantified the effect by fold change in N50 length, the effect of GC bias when 500 windows were removed was similar to the effect of repeats on assembly contiguity. Note that the number of GC windows to be removed involves both data amount and degree of GC bias. For example, a smaller amount of data at a stronger GC bias and a larger amount of data at a weaker stronger bias may lead to similar numbers of regions depleted of reads.

### Conclusions

GC bias has been a known issue in *de novo* genome assembly for more than a decade, and remains an issue in assembly using NGS data. However, a comprehensive study of the effects of GC bias on *de novo* genome assembly is still missing. As GC bias likely exists in most NGS data sets at various degrees, it is important to develop a quantitative idea of how different degrees of GC bias affect genome assembly. In this work, we conducted a systematic analysis on the effects of GC bias on genome assembly. We found that GC bias lowers the completeness of assembly when the degree of GC bias is above a threshold. The threshold likely gets smaller as the variation of GC contents increases. That is, the assembly of a genome with a greater variation of GC contents is more sensitive to GC bias. Above the threshold, assembly fragmentation because of GC bias is mainly the result of low coverage of reads at GC-poor or GC-rich regions. Increasing the amount of data rescues assembly fragmentations because of GC bias. However, the amount of data needed for a full rescue depends on the distribution of GC contents, thus is species dependent. High coverage due to GC bias also poses a harmful effect to genome assembly, e.g., more assembly errors, in general. Taken together, the uneven read coverage due to GC bias above a certain degree leads to a more fragmented and less accurate assembly. Finally, we propose an approach to accurately estimate the degree of GC bias in real NGS data without a reference genome. These pieces of information provide guidance toward a better *de novo* genome assembly in the presence of GC bias.

## Supporting Information

Figure S1 **Relationships between GC content and read coverage in the fourteen real Illumina data sets.** The fourteen data sets are from six bacterial genomes. Read coverage is normalized to the mean value, which is represented by a horizontal dashed line. A vertical dashed line denotes the mean GC content. The data points are fitted by a straight line and the slope is defined as the degree of GC bias.
(TIFF)

Figure S2 **Scatter plots of GC content and read coverage of the simulated data of bacterial genomes.** We simulated reads at 50X and 100X coverage, each at three degrees of GC bias (negative slope around −3.77, slope zero, and positive slope around slope 3.72) for three bacteria genomes: *E. coli* (A), *S. aureus* (B) and *M. tuberculosis* (C).
(TIFF)

Figure S3 **Scatter plots of GC content and read coverage of the simulated data of plant chromosomes.** For *A. thaliana* Chr. 1 (A) and *O. sativa* Chr. 5 (B), we simulated reads of 100X coverage at three degrees of GC bias (slope from −3.79 to 3.8).
(TIFF)

Figure S4 **Completeness of assemblies of three bacterial genomes by eight assemblers.** This figure is similar to Figure 4. The only difference is that we use only 20 pairs of MP reads for assemblies by ALLPATHS-LG here while 1X of MP data are used in Figure 4.
(TIFF)

Figure S5 **Completeness of the** ***E. coli*** **assemblies using reads of length 150 bp at various coverage.** This figure is similar to Figure 5. The differences are that we use read of 150 bp for assemblies and the coverage is treated as 50X, 100X, 250X and 500X. Note that the coverage values in x-axis are not scaled.
(TIFF)

Figure S6 **Distributions of coverage depths at all bases and at error bases.** Distributions of coverage depths at error bases (red curves) are compared with those at all bases (blue curves) for the eight assemblies of three bacterial genomes: *E. coli* (A), *S. aureus* (B), and *M. tuberculosis* (C). The data are simulated at a strong negative (left column), zero (middle column), and strong positive (right column) GC bias.
(TIFF)

Figure S7 **Distributions of coverage depths at the loci of three types of errors.** The three error types are missing assembly (BRK, pink), collapse of tandem repeat (DUP, green) and mis-joins (SEQ, purple) in the Velvet assemblies of the *E. coli* (A), *S. aureus* (B), and *M. tuberculosis* (C) genomes using data at a strong negative, zero, and strong positive GC bias.
(TIFF)

Figure S8 **Percentage of unaligned reference sequences in the assemblies of two plant chromosomes.** We use the eight assemblers to assemble data at a strong negative, zero, and strong positive GC bias for the two plant chromosomes: *A. thaliana* (A) and *O. sativa* (B).
(TIFF)

Figure S9 **Estimation of the degree of GC bias using reference genomes and assembled contigs.** Scatter plots of GC content and read coverage for thirteen Illumina libraries based on the known reference genomes (A) and the contigs assembled by Edena, Velvet or ABySS (B).
(TIFF)

Figure S10 **Distributions of GC contents within repeats and in whole genomes.** We use PILER to identify repeats in the five genomes: *E. coli* (A), *S. aureus* (B), and *M. tuberculosis* (C), *A. thaliana* (D), and *O. sativa* (E). The distributions of GC contents within repeats (red) are then compared with those in whole genomes (blue).
(TIFF)

Figure S11 **Effects of repeats and GC bias on genome assembly.** For each of the (A) *E. coli*, (B) *M. tuberculosis*, and (C) *S. aureus* genomes, we break the genome by removing repeats and various numbers of regions with an extreme GC content, and calculated the N50 length of the remaining sequences. A black solid line shows the genome size, and a black dashed line shows the N50 lengths in case of repeats. Blue and red curves stand for the cases where the regions with the highest and lowest GC contents are removed, respectively. We plot the blue and red curves from either the genome size or the N50 length in case of repeats to assess the effects of GC bias without and with considering the presence of repeats, respectively.
(TIFF)

Table S1 **GAGE statistics of the assemblies of E. coli by eight assemblers at three degrees of GC bias.** The simulated PE data sets of 100X coverage at three degree of GC biases are assembled by eight assemblers and the statistics of assemblies are done by GAGE.
(DOCX)

Table S2 **GAGE statistics of the assemblies of S. aureus by eight assemblers at three degrees of GC bias.** The simulated PE data sets of 100X coverage at three degree of GC biases are assembled by eight assemblers and the statistics of assemblies are done by GAGE.
(DOCX)

Table S3 **GAGE statistics of the assemblies of M. tubeculosis by eight assemblers at three degrees of GC bias.** The simulated PE data sets of 100X coverage at three degree of GC biases are assembled by eight assemblers and the statistics of assemblies are done by GAGE.
(DOCX)

Table S4 **GAGE statistics of the assemblies of A. thaliana Chr.1 by eight assemblers at three degrees of GC bias.** The simulated PE data sets of 100X coverage at three degree of GC biases are assembled by eight assemblers and the statistics of assemblies are done by GAGE.
(DOCX)

Table S5 **GAGE statistics of the assemblies of O. sativa Chr.5 by eight assemblers at three degrees of GC bias.** The simulated PE data sets of 100X coverage at three degree of GC biases are assembled by eight assemblers and the statistics of assemblies are done by GAGE.
(DOCX)

## References

[1] Schuster SC (2008) Next-generation sequencing transforms today's biology. Nat Methods 5: 16–18. 18165802 10.1038/nmeth1156

[2] Paszkiewicz K, Studholme DJ (2010) De novo assembly of short sequence reads. Brief Bioinform 11: 457–472. 20724458 10.1093/bib/bbq020

[3] Margulies M, Egholm M, Altman WE, Attiya S, Bader JS, et al (2005) Genome sequencing in microfabricated high-density picolitre reactors. Nature 437: 376–380. 16056220 10.1038/nature03959 PMC1464427

[4] Metzker ML (2010) Sequencing technologies - the next generation. Nat Rev Genet 11: 31–46. 19997069 10.1038/nrg2626

[5] Pagani I, Liolios K, Jansson J, Chen IM, Smirnova T, et al (2012) The Genomes OnLine Database (GOLD) v.4: status of genomic and metagenomic projects and their associated metadata. Nucleic Acids Res 40: D571–579. 22135293 10.1093/nar/gkr1100 PMC3245063

[6] Pop M (2009) Genome assembly reborn: recent computational challenges. Brief Bioinform 10: 354–366. 19482960 10.1093/bib/bbp026 PMC2691937

[7] Nagarajan N, Pop M (2010) Sequencing and genome assembly using next-generation technologies. Methods Mol Biol 673: 1–17. 20835789 10.1007/978-1-60761-842-3_1

[8] Smith DR, Quinlan AR, Peckham HE, Makowsky K, Tao W, et al (2008) Rapid whole-genome mutational profiling using next-generation sequencing technologies. Genome Res 18: 1638–1642. 18775913 10.1101/gr.077776.108 PMC2556265

[9] Bentley DR, Balasubramanian S, Swerdlow HP, Smith GP, Milton J, et al (2008) Accurate whole human genome sequencing using reversible terminator chemistry. Nature 456: 53–59. 18987734 10.1038/nature07517 PMC2581791

[10] Hillier LW, Marth GT, Quinlan AR, Dooling D, Fewell G, et al (2008) Whole-genome sequencing and variant discovery in C. elegans. Nat Methods 5: 183–188. 18204455 10.1038/nmeth.1179

[11] Quail MA, Kozarewa I, Smith F, Scally A, Stephens PJ, et al (2008) A large genome center's improvements to the Illumina sequencing system. Nat Methods 5: 1005–1010. 19034268 10.1038/nmeth.1270 PMC2610436

[12] Dohm JC, Lottaz C, Borodina T, Himmelbauer H (2008) Substantial biases in ultra-short read data sets from high-throughput DNA sequencing. Nucleic Acids Res 36: e105. 18660515 10.1093/nar/gkn425 PMC2532726

[13] Kozarewa I, Ning Z, Quail MA, Sanders MJ, Berriman M, et al (2009) Amplification-free Illumina sequencing-library preparation facilitates improved mapping and assembly of (G+C)-biased genomes. Nat Methods 6: 291–295. 19287394 10.1038/nmeth.1311 PMC2664327

[14] Chitsaz H, Yee-Greenbaum JL, Tesler G, Lombardo MJ, Dupont CL, et al (2011) Efficient de novo assembly of single-cell bacterial genomes from short-read data sets. Nat Biotechnol 29: 915–921. 21926975 10.1038/nbt.1966 PMC3558281

[15] Aird D, Ross MG, Chen WS, Danielsson M, Fennell T, et al (2011) Analyzing and minimizing PCR amplification bias in Illumina sequencing libraries. Genome Biol 12: R18. 21338519 10.1186/gb-2011-12-2-r18 PMC3188800

[16] Oyola SO, Otto TD, Gu Y, Maslen G, Manske M, et al (2012) Optimizing Illumina next-generation sequencing library preparation for extremely AT-biased genomes. BMC Genomics 13: 1. 22214261 10.1186/1471-2164-13-1 PMC3312816

[17] Narzisi G, Mishra B (2011) Comparing de novo genome assembly: the long and short of it. PLoS One 6: e19175. 21559467 10.1371/journal.pone.0019175 PMC3084767

[18] Zhang W, Chen J, Yang Y, Tang Y, Shang J, et al (2011) A practical comparison of de novo genome assembly software tools for next-generation sequencing technologies. PLoS One 6: e17915. 21423806 10.1371/journal.pone.0017915 PMC3056720

[19] Lin Y, Li J, Shen H, Zhang L, Papasian CJ, et al (2011) Comparative studies of de novo assembly tools for next-generation sequencing technologies. Bioinformatics 27: 2031–2037. 21636596 10.1093/bioinformatics/btr319 PMC3137213

[20] Sayers EW, Barrett T, Benson DA, Bolton E, Bryant SH, et al (2012) Database resources of the National Center for Biotechnology Information. Nucleic Acids Res 40: D13–25. 22140104 10.1093/nar/gkr1184 PMC3245031

[21] novocraft website. Available: http://www.novocraft.com/main/page.php?s=novoalign. Accessed 2010.

[22] Ruffalo M, LaFramboise T, Koyuturk M (2011) Comparative analysis of algorithms for next-generation sequencing read alignment. Bioinformatics 27: 2790–2796. 21856737 10.1093/bioinformatics/btr477

[23] Edgar RC, Myers EW (2005) PILER: identification and classification of genomic repeats. Bioinformatics 21 Suppl 1i152–158. 15961452 10.1093/bioinformatics/bti1003

[24] Butler J, MacCallum I, Kleber M, Shlyakhter IA, Belmonte MK, et al (2008) ALLPATHS: de novo assembly of whole-genome shotgun microreads. Genome Res 18: 810–820. 18340039 10.1101/gr.7337908 PMC2336810

[25] Maccallum I, Przybylski D, Gnerre S, Burton J, Shlyakhter I, et al (2009) ALLPATHS 2: small genomes assembled accurately and with high continuity from short paired reads. Genome Biol 10: R103. 19796385 10.1186/gb-2009-10-10-r103 PMC2784318

[26] Simpson JT, Wong K, Jackman SD, Schein JE, Jones SJ, et al (2009) ABySS: a parallel assembler for short read sequence data. Genome Res 19: 1117–1123. 19251739 10.1101/gr.089532.108 PMC2694472

[27] Schmidt B, Sinha R, Beresford-Smith B, Puglisi SJ (2009) A fast hybrid short read fragment assembly algorithm. Bioinformatics 25: 2279–2280. 19535537 10.1093/bioinformatics/btp374

[28] Li R, Zhu H, Ruan J, Qian W, Fang X, et al (2010) De novo assembly of human genomes with massively parallel short read sequencing. Genome Res 20: 265–272. 20019144 10.1101/gr.097261.109 PMC2813482

[29] Warren RL, Sutton GG, Jones SJ, Holt RA (2007) Assembling millions of short DNA sequences using SSAKE. Bioinformatics 23: 500–501. 17158514 10.1093/bioinformatics/btl629 PMC7109930

[30] Zerbino DR, Birney E (2008) Velvet: algorithms for de novo short read assembly using de Bruijn graphs. Genome Res 18: 821–829. 18349386 10.1101/gr.074492.107 PMC2336801

[31] Zerbino DR, McEwen GK, Margulies EH, Birney E (2009) Pebble and rock band: heuristic resolution of repeats and scaffolding in the velvet short-read de novo assembler. PLoS One 4: e8407. 20027311 10.1371/journal.pone.0008407 PMC2793427

[32] Salzberg SL, Phillippy AM, Zimin AV, Puiu D, Magoc T, et al.. (2011) GAGE: A critical evaluation of genome assemblies and assembly algorithms. Genome Res. 10.1101/gr.131383.111 PMC3290791 22147368

[33] Kurtz S, Phillippy A, Delcher AL, Smoot M, Shumway M, et al (2004) Versatile and open software for comparing large genomes. Genome Biol 5: R12. 14759262 10.1186/gb-2004-5-2-r12 PMC395750

[34] Benjamini Y, Speed TP (2012) Summarizing and correcting the GC content bias in high-throughput sequencing. Nucleic Acids Res 40: e72. 22323520 10.1093/nar/gks001 PMC3378858

[35] Arezi B, Xing W, Sorge JA, Hogrefe HH (2003) Amplification efficiency of thermostable DNA polymerases. Anal Biochem 321: 226–235. 14511688 10.1016/s0003-2697(03)00465-2

[36] Ralser M, Querfurth R, Warnatz HJ, Lehrach H, Yaspo ML, et al (2006) An efficient and economic enhancer mix for PCR. Biochem Biophys Res Commun 347: 747–751. 16842759 10.1016/j.bbrc.2006.06.151

[37] Hube F, Reverdiau P, Iochmann S, Gruel Y (2005) Improved PCR method for amplification of GC-rich DNA sequences. Mol Biotechnol 31: 81–84. 16118416 10.1385/MB:31:1:081

[38] Mamedov TG, Pienaar E, Whitney SE, TerMaat JR, Carvill G, et al (2008) A fundamental study of the PCR amplification of GC-rich DNA templates. Comput Biol Chem 32: 452–457. 18760969 10.1016/j.compbiolchem.2008.07.021 PMC2727727

[39] Suzuki MT, Giovannoni SJ (1996) Bias caused by template annealing in the amplification of mixtures of 16S rRNA genes by PCR. Appl Environ Microbiol 62: 625–630. 8593063 10.1128/aem.62.2.625-630.1996 PMC167828

[40] Koboldt DC, Ding L, Mardis ER, Wilson RK (2010) Challenges of sequencing human genomes. Brief Bioinform 11: 484–498. 20519329 10.1093/bib/bbq016 PMC2980933

[41] Golovko G, Khanipov K, Rojas M, Martinez-Alcantara A, Howard JJ, et al (2012) Slim-Filter: an interactive windows-based application for illumina genome analyzer data assessment and manipulation. BMC Bioinformatics 13: 166. 22800377 10.1186/1471-2105-13-166 PMC3505481

[42] Shintani M, Matsumoto T, Yoshikawa H, Yamane H, Ohkuma M, et al (2011) DNA rearrangement has occurred in the carbazole-degradative plasmid pCAR1 and the chromosome of its unsuitable host, Pseudomonas fluorescens Pf0–1. Microbiology 157: 3405–3416. 21948045 10.1099/mic.0.053280-0

[43] Fisher S, Barry A, Abreu J, Minie B, Nolan J, et al (2011) A scalable, fully automated process for construction of sequence-ready human exome targeted capture libraries. Genome Biol 12: R1. 21205303 10.1186/gb-2011-12-1-r1 PMC3091298

