# Supplementary figures and images for: Effects of GC Bias in Next-Generation-Sequencing Data on De Novo Genome Assembly
Source: PLoS One. 2013 Apr 29;8(4):e62856. doi: 10.1371/journal.pone.0062856 (PMC3639258; doi:10.1371/journal.pone.0062856)

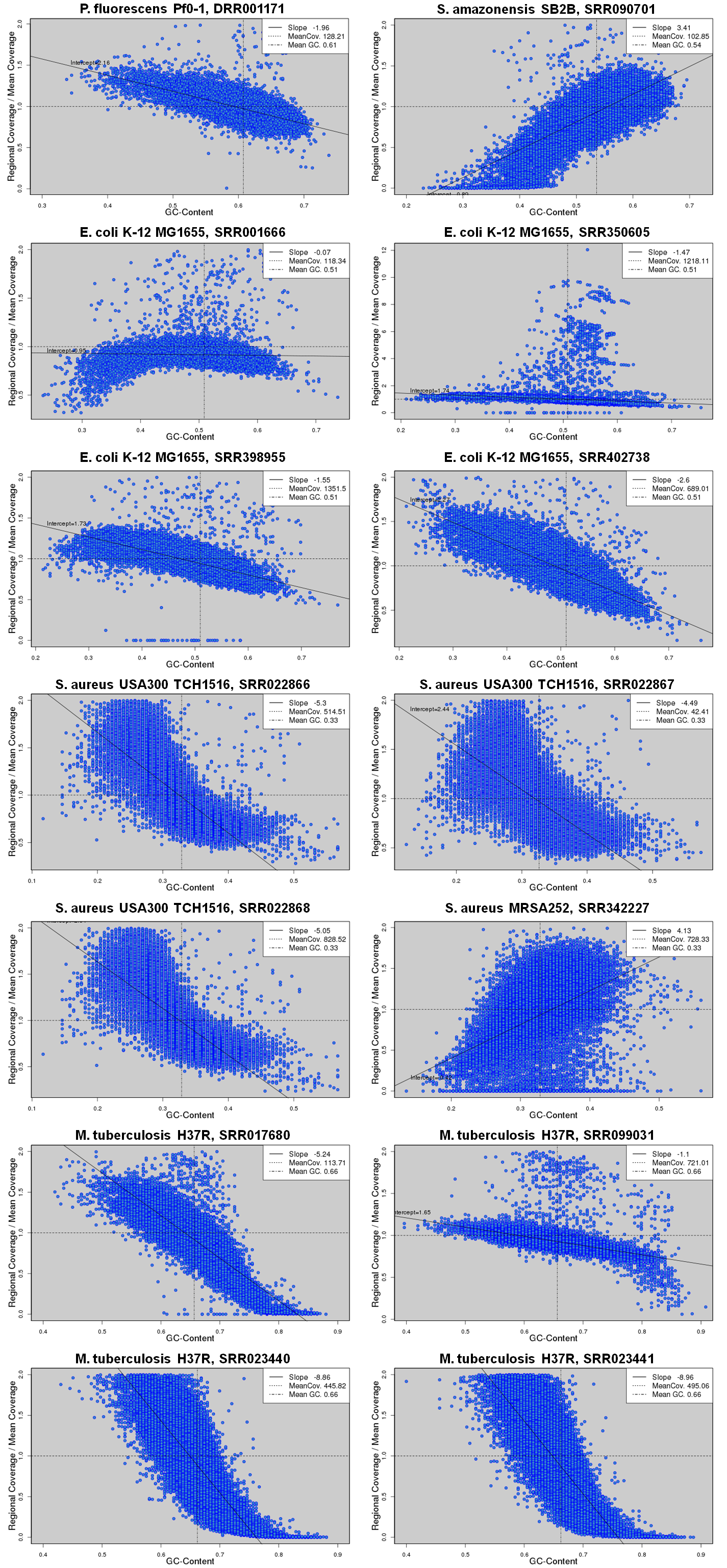

Supplement: Figure S1 — Relationships between GC content and read coverage in the fourteen real Illumina data sets. The fourteen data sets are from six bacterial genomes. Read coverage is normalized to the mean value, which is represented by a horizontal dashed line. A vertical dashed line denotes the mean GC content. The data points are fitted by a straight line and the slope is defined as the degree of GC bias. (TIFF) [file pone.0062856.s001.tiff]

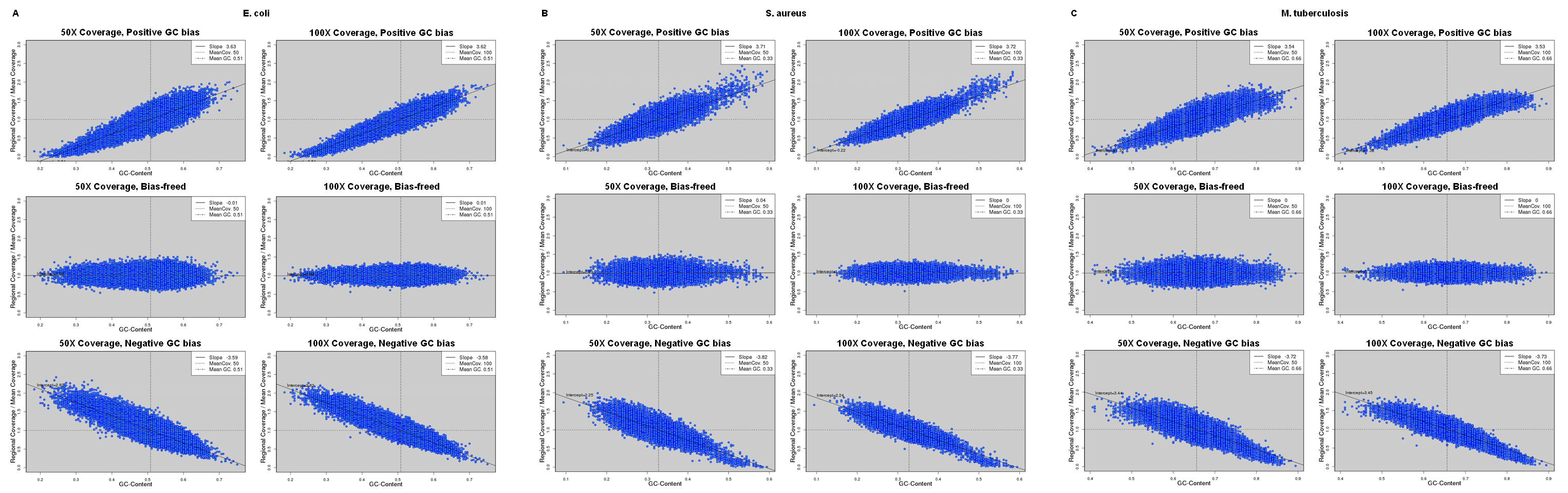

Supplement: Figure S2 — Scatter plots of GC content and read coverage of the simulated data of bacterial genomes. We simulated reads at 50X and 100X coverage, each at three degrees of GC bias (negative slope around −3.77, slope zero, and positive slope around slope 3.72) for three bacteria genomes: E. coli (A), S. aureus (B) and M. tuberculosis (C). (TIFF) [file pone.0062856.s002.tiff]

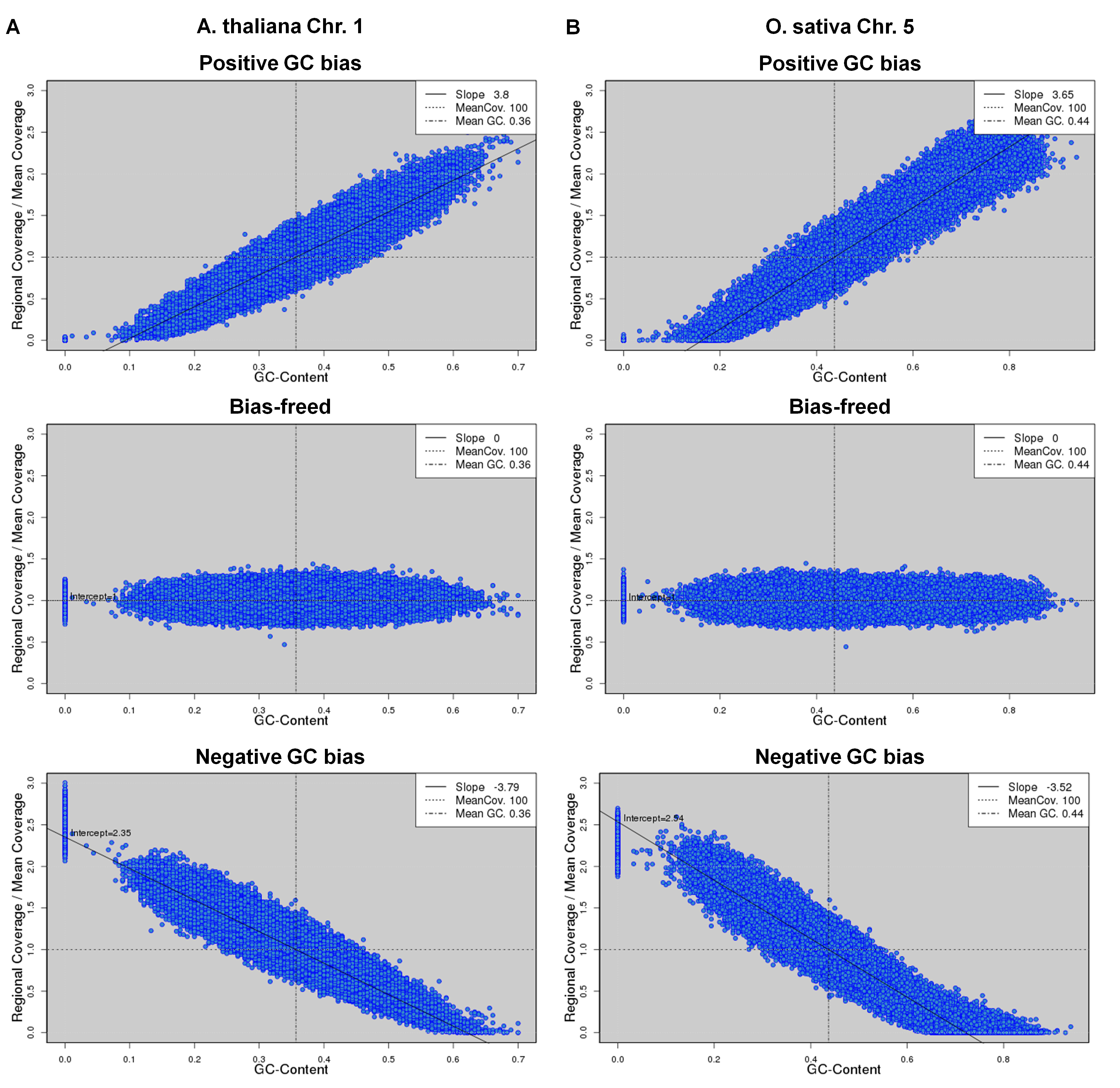

Supplement: Figure S3 — Scatter plots of GC content and read coverage of the simulated data of plant chromosomes. For A. thaliana Chr. 1 (A) and O. sativa Chr. 5 (B), we simulated reads of 100X coverage at three degrees of GC bias (slope from −3.79 to 3.8). (TIFF) [file pone.0062856.s003.tiff]

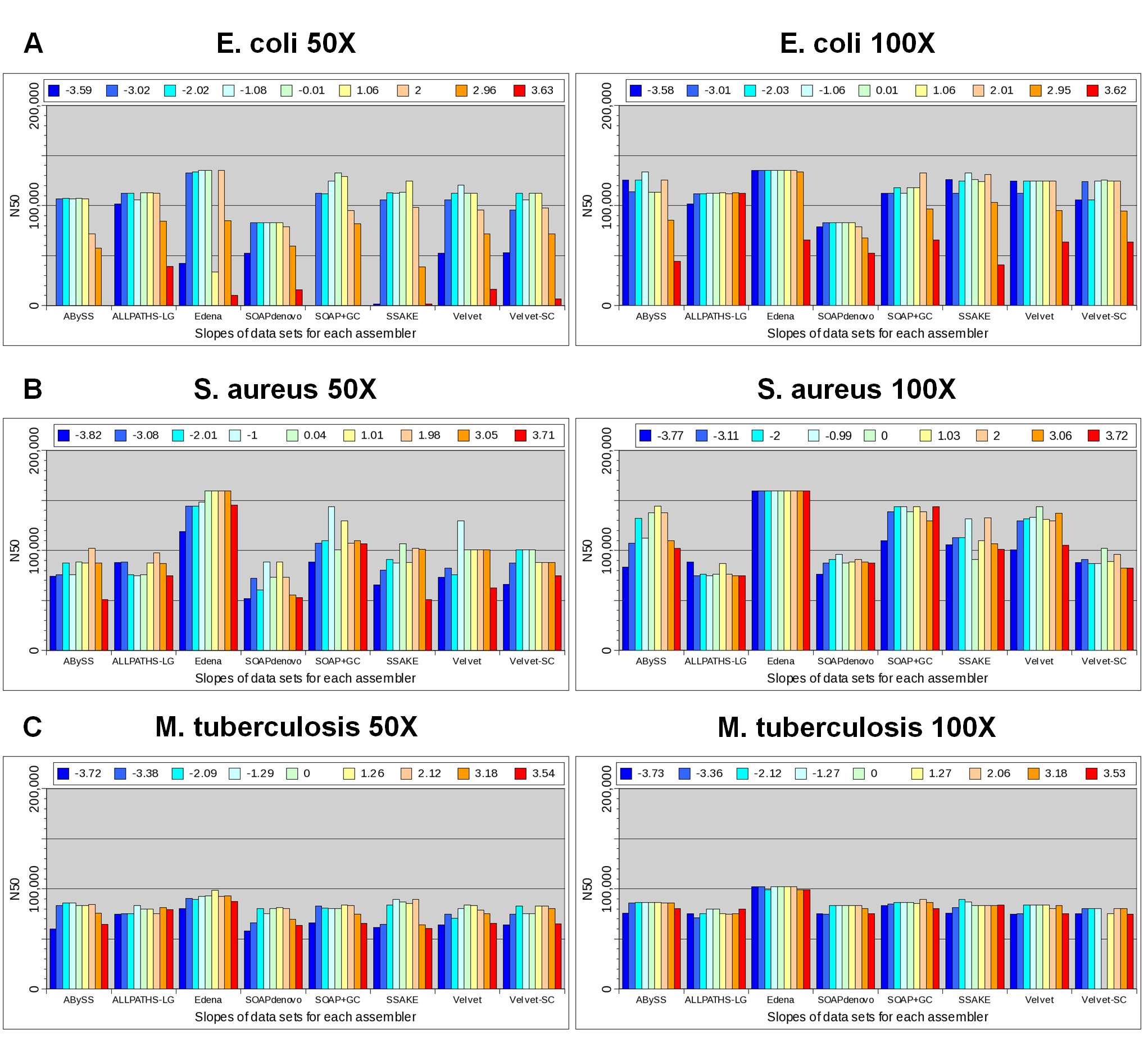

Supplement: Figure S4 — Completeness of assemblies of three bacterial genomes by eight assemblers. This figure is similar to Figure 4. The only difference is that we use only 20 pairs of MP reads for assemblies by ALLPATHS-LG here while 1X of MP data are used in Figure 4. (TIFF) [file pone.0062856.s004.tiff]

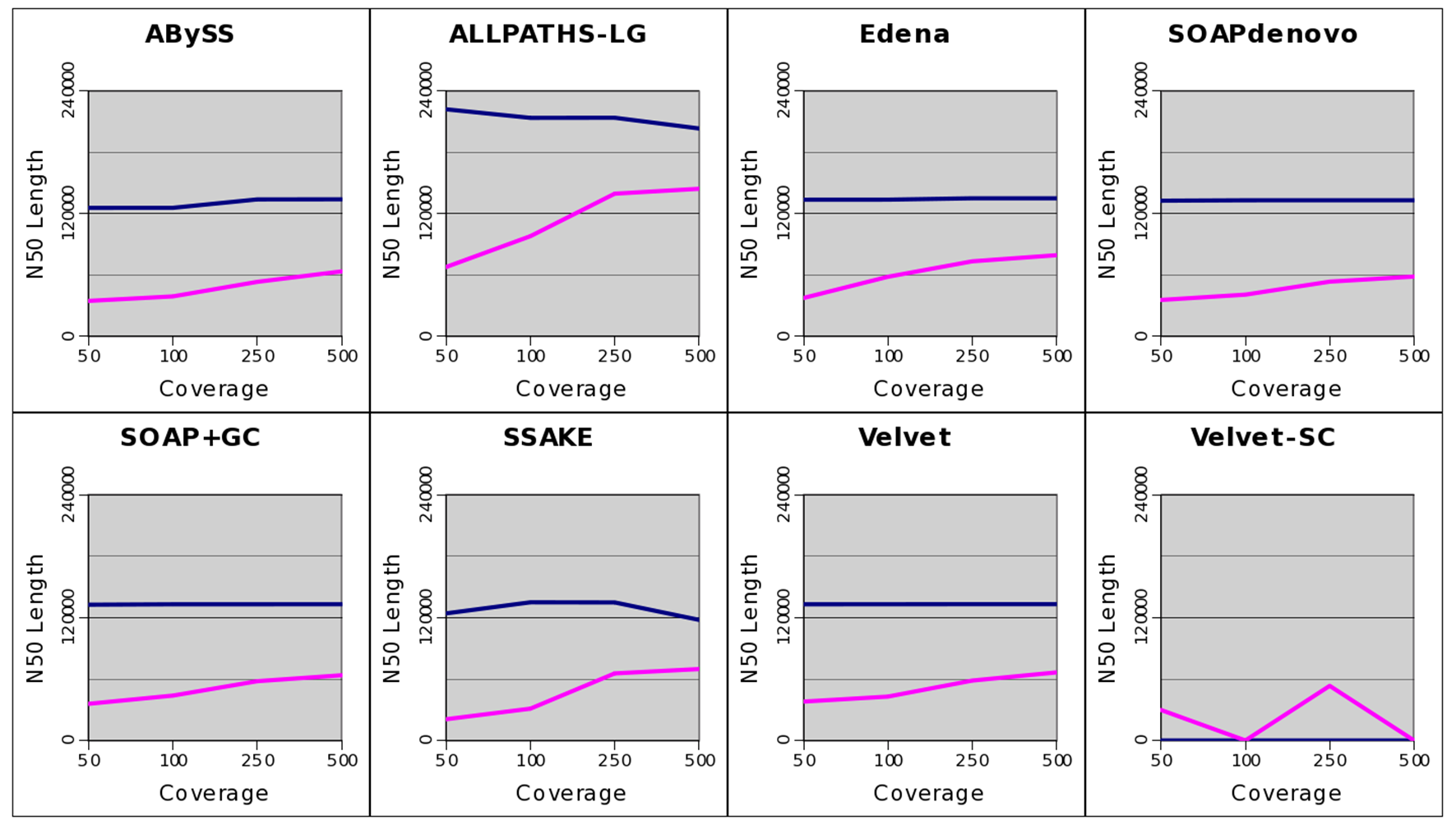

Supplement: Figure S5 — Completeness of the E. coli assemblies using reads of length 150 bp at various coverage. This figure is similar to Figure 5. The differences are that we use read of 150 bp for assemblies and the coverage is treated as 50X, 100X, 250X and 500X. Note that the coverage values in x-axis are not scaled. (TIFF) [file pone.0062856.s005.tiff]

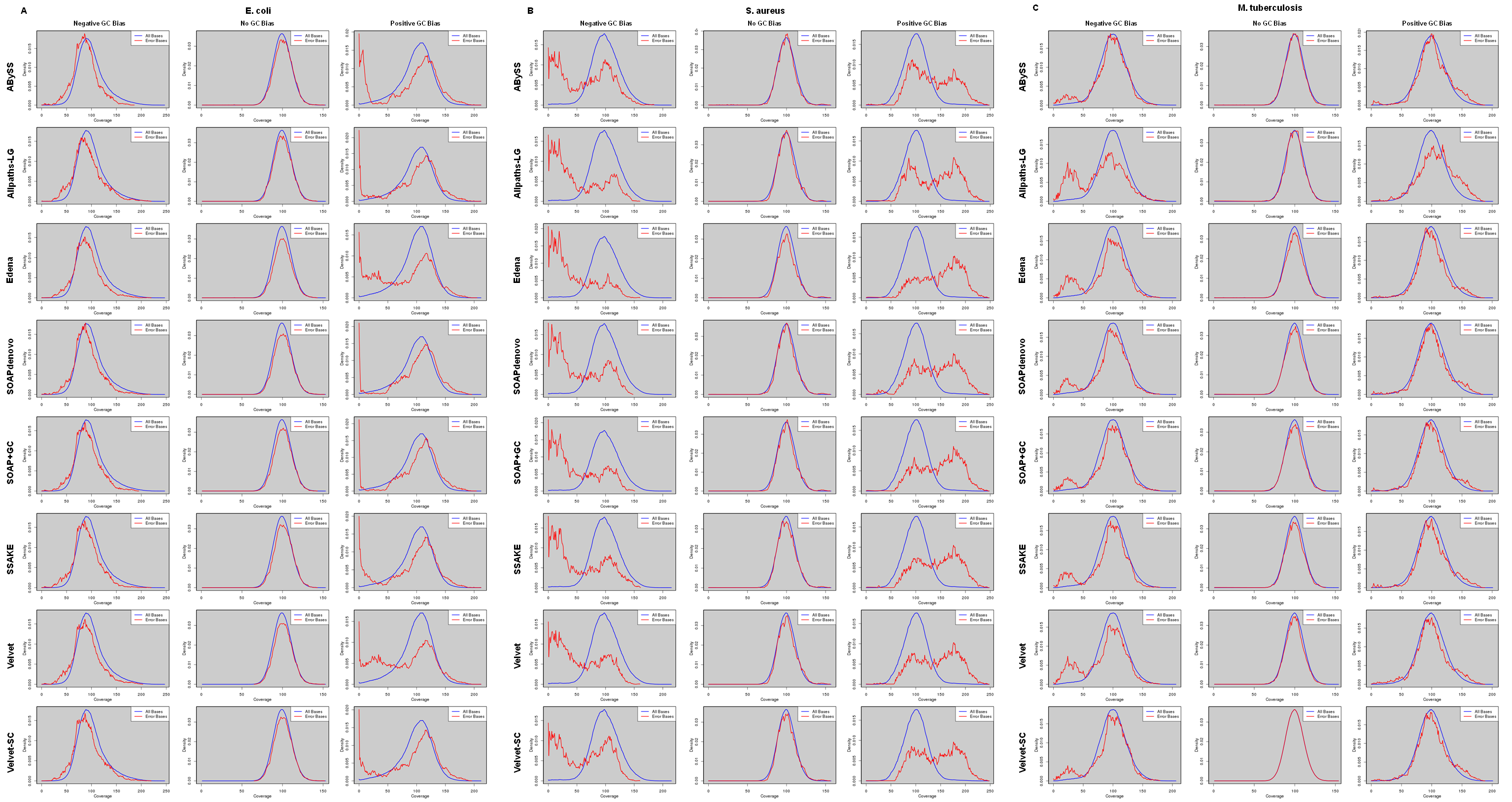

Supplement: Figure S6 — Distributions of coverage depths at all bases and at error bases. Distributions of coverage depths at error bases (red curves) are compared with those at all bases (blue curves) for the eight assemblies of three bacterial genomes: E. coli (A), S. aureus (B), and M. tuberculosis (C). The data are simulated at a strong negative (left column), zero (middle column), and strong positive (right column) GC bias. (TIFF) [file pone.0062856.s006.tiff]

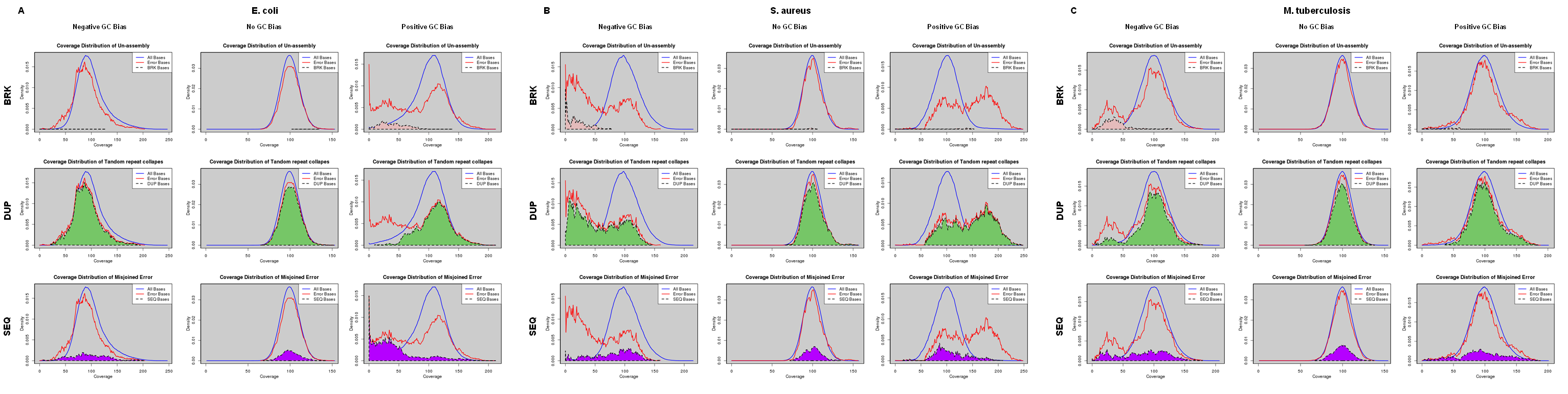

Supplement: Figure S7 — Distributions of coverage depths at the loci of three types of errors. The three error types are missing assembly (BRK, pink), collapse of tandem repeat (DUP, green) and mis-joins (SEQ, purple) in the Velvet assemblies of the E. coli (A), S. aureus (B), and M. tuberculosis (C) genomes using data at a strong negative, zero, and strong positive GC bias. (TIFF) [file pone.0062856.s007.tiff]

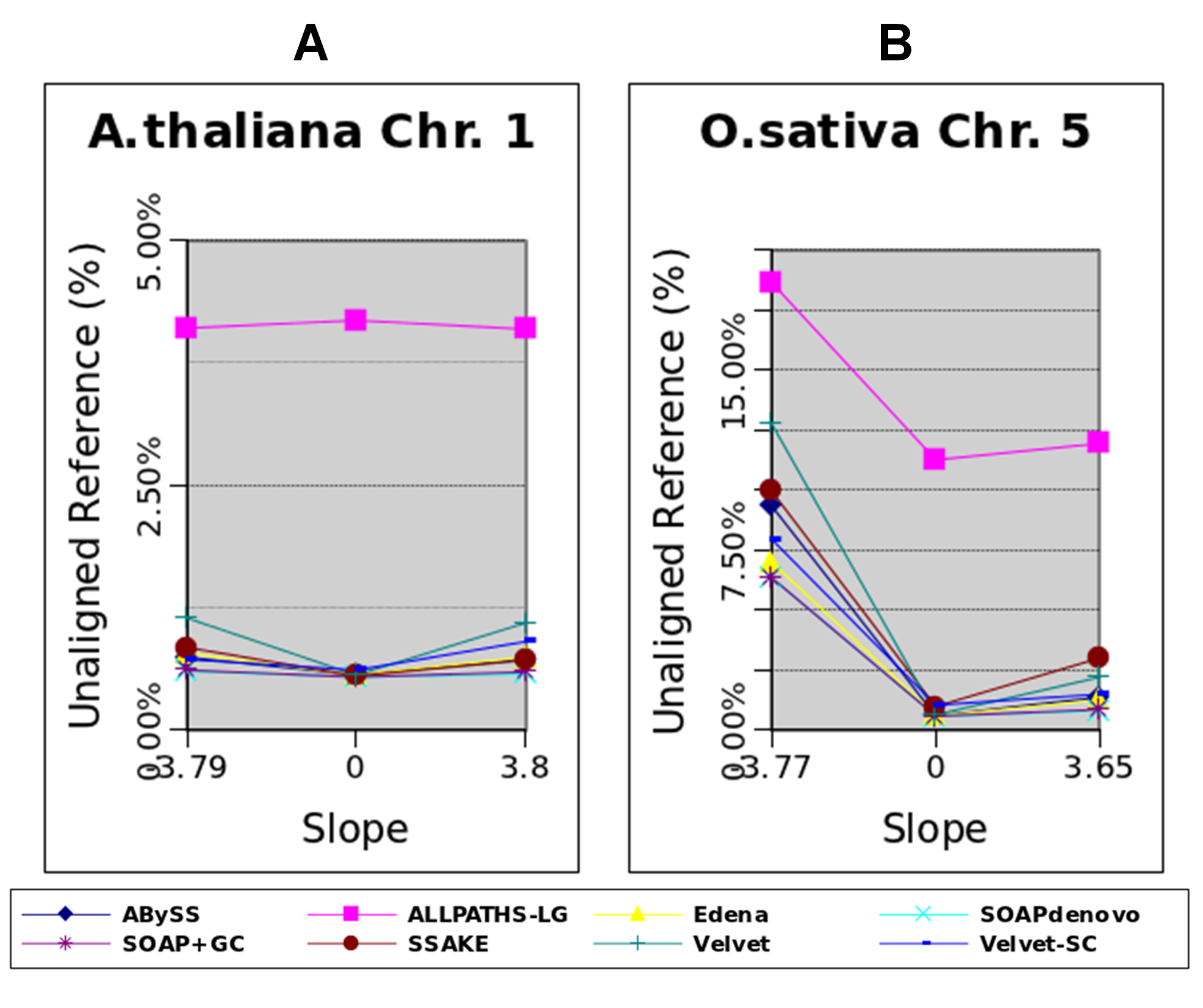

Supplement: Figure S8 — Percentage of unaligned reference sequences in the assemblies of two plant chromosomes. We use the eight assemblers to assemble data at a strong negative, zero, and strong positive GC bias for the two plant chromosomes: A. thaliana (A) and O. sativa (B). (TIFF) [file pone.0062856.s008.tiff]

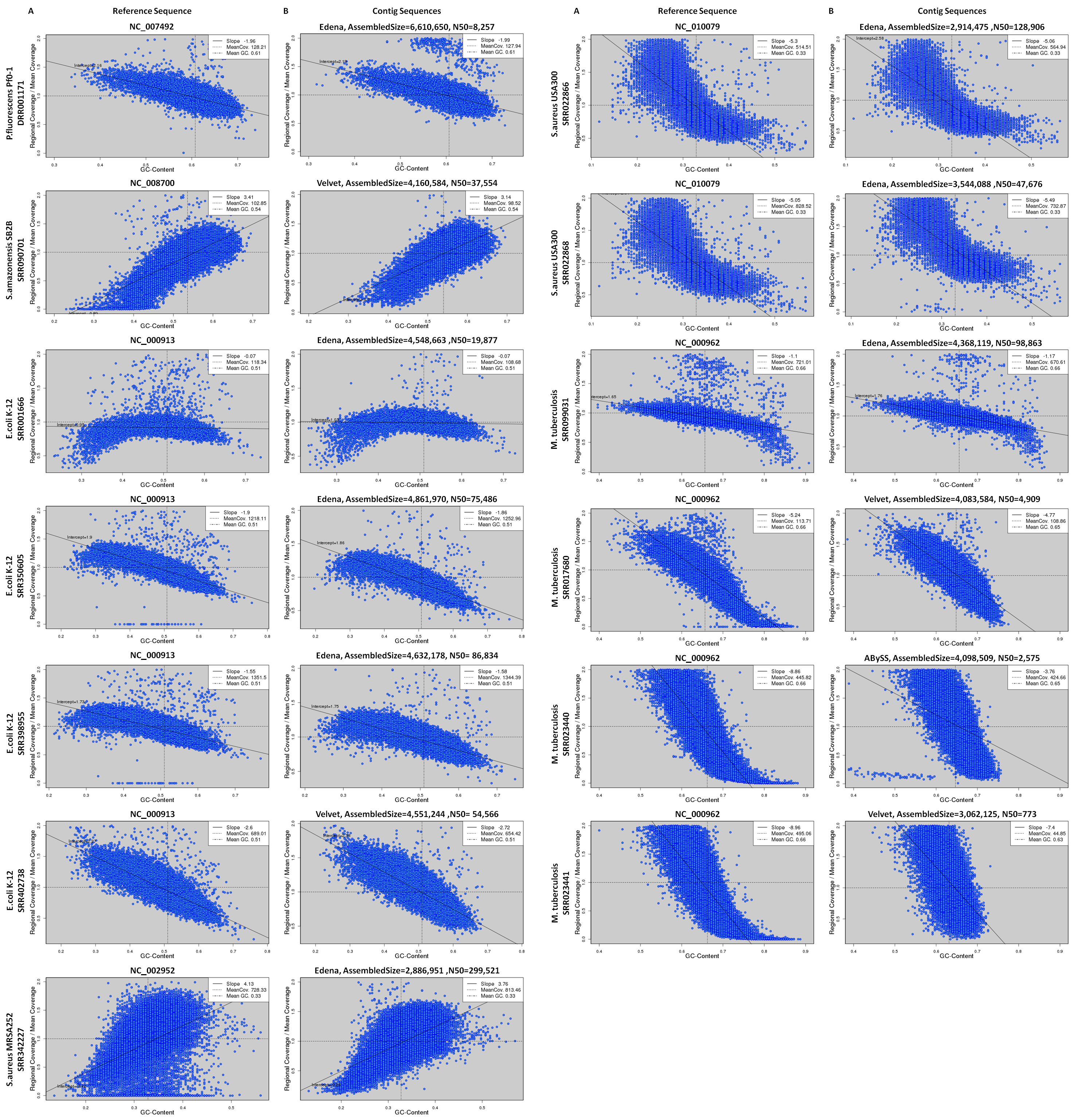

Supplement: Figure S9 — Estimation of the degree of GC bias using reference genomes and assembled contigs. Scatter plots of GC content and read coverage for thirteen Illumina libraries based on the known reference genomes (A) and the contigs assembled by Edena, Velvet or ABySS (B). (TIFF) [file pone.0062856.s009.tiff]

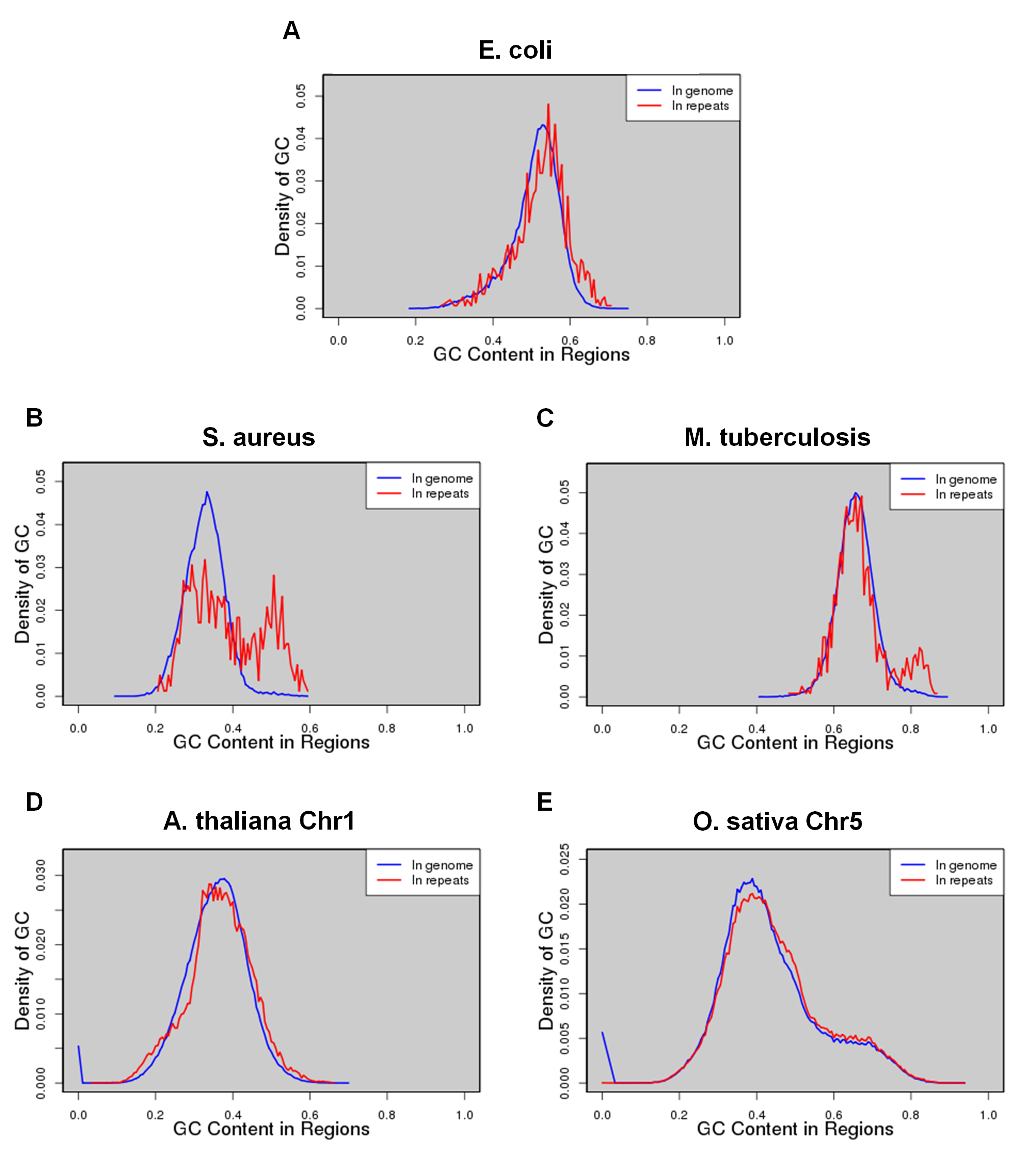

Supplement: Figure S10 — Distributions of GC contents within repeats and in whole genomes. We use PILER to identify repeats in the five genomes: E. coli (A), S. aureus (B), and M. tuberculosis (C), A. thaliana (D), and O. sativa (E). The distributions of GC contents within repeats (red) are then compared with those in whole genomes (blue). (TIFF) [file pone.0062856.s010.tiff]

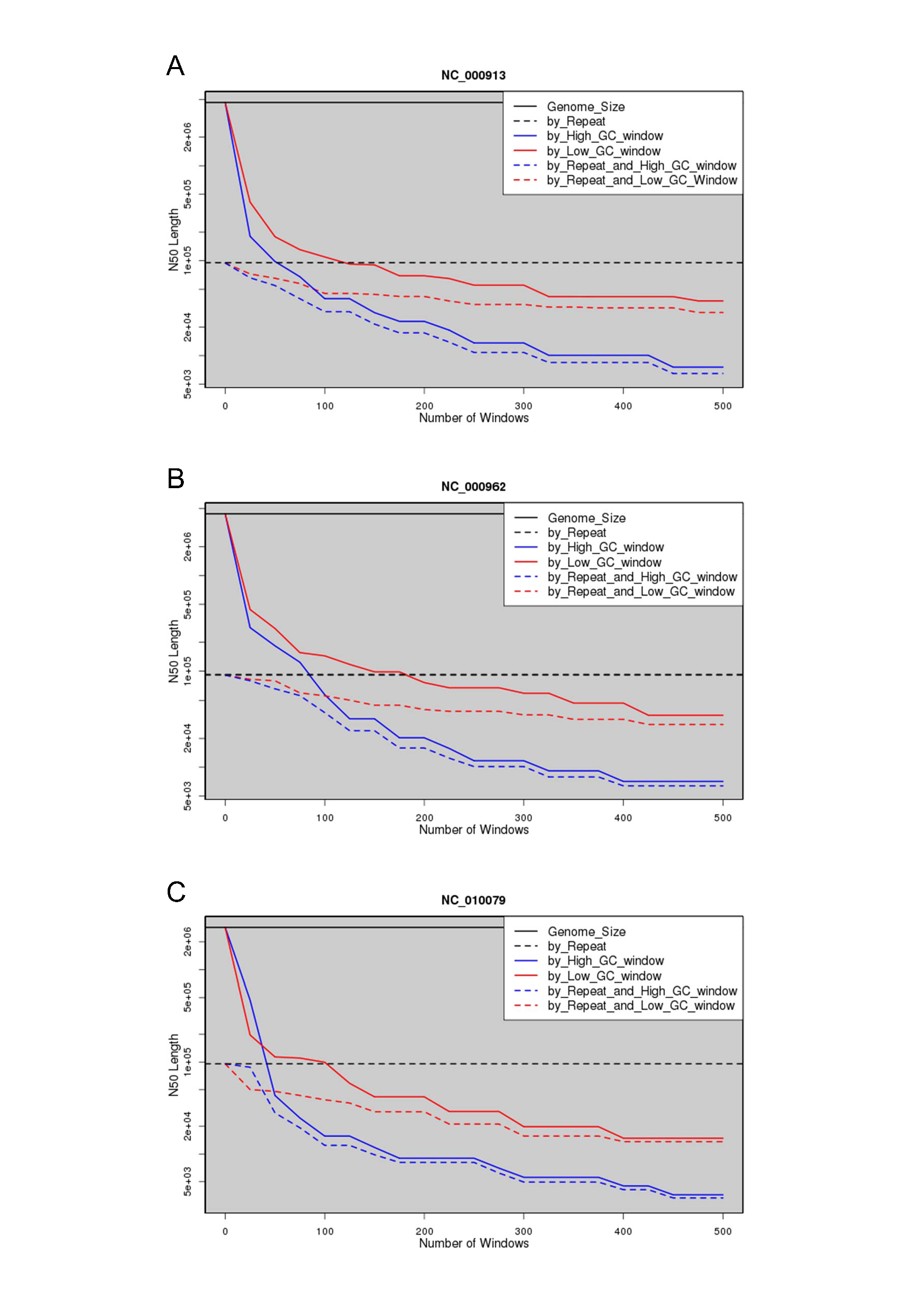

Supplement: Figure S11 — Effects of repeats and GC bias on genome assembly. For each of the (A) E. coli, (B) M. tuberculosis, and (C) S. aureus genomes, we break the genome by removing repeats and various numbers of regions with an extreme GC content, and calculated the N50 length of the remaining sequences. A black solid line shows the genome size, and a black dashed line shows the N50 lengths in case of repeats. Blue and red curves stand for the cases where the regions with the highest and lowest GC contents are removed, respectively. We plot the blue and red curves from either the genome size or the N50 length in case of repeats to assess the effects of GC bias without and with considering the presence of repeats, respectively. (TIFF) [file pone.0062856.s011.tiff]
