# Supplementary material for: Effects of GC Bias in Next-Generation-Sequencing Data on De Novo Genome Assembly
Source: PLoS One. 2013 Apr 29;8(4):e62856. doi: 10.1371/journal.pone.0062856 (PMC3639258; doi:10.1371/journal.pone.0062856)
Supplement: Table S5 — GAGE statistics of the assemblies of O. sativa Chr.5 by eight assemblers at three degrees of GC bias. The simulated PE data sets of 100X coverage at three degree of GC biases are assembled by eight assemblers and the statistics of assemblies are done by GAGE. (DOCX) [file pone.0062856.s016.docx]

**Table S5. GAGE statistics of the assemblies of *O. sativa*** **Chr.5 by eight assemblers at three degrees of GC bias.**

| **Assembler** | **Slope** | **Assembled Bases (%)** | **Unaligned Ref (%)** | **Unaligned Asm (%)** | **Duplicated Ref (%)** | **Compressed Ref (%)** | **SNPs** | **Indels <5bp** | **Indels >=5** | **Inver -sion** | **Reloca -tion** |
| --- | --- | --- | --- | --- | --- | --- | --- | --- | --- | --- | --- |
| ALLPATHS-LG | 3.65 | 83.17% | 11.93% | 0.00% | 0.08% | 6.33% | 2,569 | 374 | 131 | 10 | 11 |
|  | 0 | 83.84% | 11.25% | 0.00% | 0.04% | 6.39% | 2,392 | 229 | 95 | 5 | 21 |
|  | -3.77 | 76.95% | 18.64% | 0.00% | 0.12% | 5.76% | 2,311 | 256 | 103 | 12 | 13 |
| ABySS | 3.65 | 101.19% | 1.36% | 0.00% | 4.84% | 7.26% | 4,234 | 355 | 23 | 20 | 14 |
|  | 0 | 103.43% | 0.58% | 0.00% | 4.47% | 5.36% | 2,913 | 300 | 24 | 9 | 7 |
|  | -3.77 | 91.50% | 9.33% | 0.00% | 3.72% | 6.92% | 3,398 | 338 | 49 | 9 | 11 |
| Velvet | 3.65 | 89.68% | 2.19% | 0.01% | 0.61% | 14.05% | 2,010 | 199 | 51 | 57 | 52 |
|  | 0 | 93.71% | 0.63% | 0.01% | 0.36% | 10.57% | 513 | 101 | 27 | 2 | 7 |
|  | -3.77 | 82.85% | 12.76% | 4.66% | 0.45% | 12.25% | 2,012 | 227 | 58 | 39 | 25 |
| Velvet-SC | 3.65 | 90.07% | 1.48% | 0.13% | 0.54% | 14.12% | 3,650 | 874 | 388 | 229 | 268 |
|  | 0 | 90.66% | 1.03% | 0.21% | 0.64% | 13.46% | 4,957 | 1,428 | 593 | 83 | 151 |
|  | -3.77 | 84.06% | 7.94% | 0.19% | 0.54% | 12.50% | 4,974 | 1,038 | 476 | 251 | 370 |
| SOAPdenovo | 3.65 | 104.95% | 0.80% | 2.71% | 6.06% | 7.52% | 111 | 5 | 1 | 2 | 0 |
|  | 0 | 105.26% | 0.57% | 0.03% | 7.89% | 5.85% | 60 | 1 | 0 | 0 | 1 |
|  | -3.77 | 97.05% | 6.33% | 0.03% | 6.77% | 7.06% | 132 | 4 | 1 | 1 | 2 |
| SOAP+GC | 3.65 | 94.33% | 0.84% | 0.02% | 0.57% | 9.95% | 792 | 69 | 34 | 7 | 5 |
|  | 0 | 98.14% | 0.57% | 0.03% | 1.81% | 6.77% | 582 | 66 | 58 | 2 | 5 |
|  | -3.77 | 89.86% | 6.34% | 0.02% | 0.73% | 8.46% | 657 | 32 | 48 | 4 | 6 |
| Edena | 3.65 | 96.41% | 1.24% | 0.00% | 0.83% | 6.45% | 152 | 18 | 0 | 5 | 6 |
|  | 0 | 97.44% | 0.58% | 0.00% | 0.82% | 5.86% | 76 | 7 | 0 | 1 | 1 |
|  | -3.77 | 90.21% | 6.99% | 0.00% | 0.92% | 6.94% | 350 | 65 | 0 | 8 | 7 |
| SSAKE | 3.65 | 92.38% | 2.99% | 0.01% | 2.43% | 12.02% | 3,778 | 296 | 6 | 66 | 58 |
|  | 0 | 93.37% | 0.97% | 0.02% | 1.67% | 11.89% | 4,223 | 327 | 6 | 87 | 67 |
|  | -3.77 | 84.59% | 9.94% | 0.01% | 1.80% | 11.58% | 3,001 | 197 | 6 | 55 | 42 |

The simulated PE data sets of 100X coverage at three degree of GC biases are assembled by eight assemblers and the statistics of assemblies are done by GAGE.
